# Development of a Questionnaire for the Search for Occupational Causes in Patients with Non-Hodgkin Lymphoma: The RHELYPRO Study

**DOI:** 10.3390/ijerph18084008

**Published:** 2021-04-11

**Authors:** Mireille Matrat, Murielle Gain, Corinne Haioun, Fabien Le Bras, Catherine Nisse, Franck Morschhauser, Bénédicte Clin, Isabelle Baldi, Catherine Verdun-Esquer, Robert Garnier, Hervé Laborde-Castérot, Fabrice Hérin, Yolande Esquirol, Pascal Andujar, Milia Belacel, Christos Chouaïd, Claire Chauvet, Gérard Lasfargues, Jean-Claude Pairon

**Affiliations:** 1 Service des Pathologies Professionnelles et de l’Environnement, CHI Créteil, F-94010 Créteil, France; mireille.matrat@chicreteil.fr (M.M.); muriellegain2000@yahoo.fr (M.G.); pascal.andujar@chicreteil.fr (P.A.); 2 INSERM, IMRB, Paris Est Créteil University, F-94010 Créteil, France; corinne.haioun@aphp.fr; 3 AP-HP, Hôpital Henri Mondor, Unité Hémopathies Lymphoïdes, F-94010 Créteil, France; fabien.le-bras@aphp.fr; 4 CHU Lille, Institut Pasteur de Lille, Université de Lille, 4483-IMPECS-Impact de l’environnement Chimique sur la Santé Humaine, F-59000 Lille, France; catherine.nisse@chru-lille.fr; 5 ULR 7365-GRITA-Groupe de Recherche sur les Formes Injectables et les Technologies Associées, Université de Lille, CHU Lille, F-59000 Lille, France; franck.morschhauser@chru-lille.fr; 6 Centre Régional de Pathologies Professionnelles et Environnementales, CHU de Caen, INSERM U1086, Cancers et Préventions, Université de Caen Normandie, F-14033 Caen, France; clin-b@chu-caen.fr; 7 Service Santé Travail Environnement, CHU Bordeaux, INSERM U1219, EPICENE, Bordeaux University, F-33076 Bordeaux, France; isabelle.baldi@u-bordeaux.fr (I.B.); catherine.verdun-esquer@chu-bordeaux.fr (C.V.-E.); 8 Centre Antipoison de Paris, Consultation de Pathologie Professionnelle et de l’Environnement, AP-HP, Nord-Université de Paris, Hôpital Lariboisière-Fernand Widal-St Louis, F-75475 Paris, France; robert.garnier@aphp.fr (R.G.); herve.laborde-casterot@aphp.fr (H.L.-C.); 9 Centre Régional de Pathologies Professionnelles et Environnementales, CHU Toulouse, F-31059 Toulouse, France; herin.f@chu-toulouse.fr (F.H.); esquirol.y@chu-toulouse.fr (Y.E.); 10 Institut Santé-Travail Paris-Est, CHI Créteil, F-94010 Créteil, France; milia.belacel@chicreteil.fr; 11 Centre de Recherche Clinique, CHI Créteil, F-94010 Créteil, France; christos.chouaid@chicreteil.fr; 12 Anses, Agence Nationale de Sécurité Sanitaire de l’alimentation, de l’environnement et du Travail, F-94700 Maisons-Alfort, France; claire.chauvet@anses.fr (C.C.); gerard.lasfargues@anses.fr (G.L.)

**Keywords:** non-Hodgkin lymphoma, occupational disease, exposure to solvents, exposure to pesticides, workers’ compensation

## Abstract

Non-Hodgkin lymphoma (NHL), multiple myeloma and chronic lymphocytic leukemia are possibly related to environmental and/or occupational exposure. The primary objective of this study was to develop a questionnaire for screening patients with these blood disorders who might benefit from a specialized consultation for possible recognition of the disease as an occupational disease. The study included 205 subjects (male gender, 67.3%; mean age, 60 years; NHL, 78.5%). The questionnaire performed very satisfactorily in identifying the exposures most frequently retained by experts for their potential involvement in the occurrence of NHL. Its sensitivity and specificity in relation to the final expertise were 96% and 96% for trichloroethylene, 85% and 82% for benzene, 78% and 87% for solvents other than trichloroethylene and dichloromethane, 87% and 95% for pesticides, respectively. Overall, 15% of the subjects were invited to ask National Social Insurance for compensation as occupational disease. These declarations concerned exposure to pesticides (64%), solvents (trichloroethylene: 29%; benzene: 18%; other than chlorinated solvents: 18%) and sometimes multiple exposures. In conclusion, this questionnaire appears as a useful tool to identify NHL patients for a specialized consultation, in order to ask for compensation for occupational disease.

## 1. Introduction

The high prevalence and the increase in the incidence of non-Hodgkin lymphoma (NHL), multiple myeloma (MM) and chronic lymphocytic leukemia (CLL) remain a major concern in 2020 [1]. In France, there were 10,224 incident cases for all NHL subtypes, excluding CLL and MM [1,2]. In Europe, in 2020, the number of new NHL cases was 123,000 and the number of deaths was 49,700 with a 5-year prevalence equal to 389,000 cases [3]. For hematologists, MM and CLL are considered as “lymphomas”. Therefore, these three disorders (NHL, CLL, MM) will be grouped under the term “lymphomas” in this article.

These conditions may be related to environmental and/or occupational exposure to various substances or agents and are therefore likely to be recognized as occupational diseases. Although the impact of occupational factors in the risk of NHL occurrence is still unclear, some occupations have been associated with this risk, including farmers. The main hypothesis underlies on the involvement of pesticide exposures in the occurrence of non-Hodgkin lymphoma [4,5].

The objective of the RHELYPRO study (for “Repérage des HEmopathies LYmphoïdes d’origine PROfessionnelle”) was to build an occupational questionnaire to investigate exposures throughout the career of workers and to compare the answers to assessments carried out by experts. The investigator completed the questionnaire with data collected during the consultation of patients with “lymphoma” (i.e., NHL or MM or CLL). This questionnaire was a tool for the clinicians to select patients they should refer to a specialized consultation in order to further evaluate exposures and potentially apply for recognition of occupational disease.

## 2. Materials and Methods

### 2.1. Study Design and Investigator Centers

The RHELYPRO study was a multicenter study performed in seven centers in France: a hematology department (CHU Henri-Mondor, Créteil; Lymphoid malignancies Unit) working with a specialized occupational disease consultation (CHI of Créteil; Occupational and Environmental Disease Department) and five specialized occupational disease consultation centers in relationship with the hematology department of the same university hospital: Bordeaux, Caen, Lille, Toulouse and Paris (Lariboisière Fernand-Widal). Inclusions started from March 2017 to January 2018, depending on the center, and were discontinued in March 2019.

The study conformed to the principles of the Declaration of Helsinki and Good Clinical Practice Guidelines. It was approved by Ethics Committee (“CPP Sud Ouest et Outre Mer II”; 2 March 2017). Patients were orally informed of the objectives of the study. The study was registered at ClinicalTrials.gov (accessed on 10 April 2021) NCT03316209.

### 2.2. Study Population

Patients, male or female, eligible for the questionnaire had to be diagnosed as NHL, CLL or MM within 2 years at the time of inclusion. This condition must not have occurred in the context of HIV infection or following transplantation. Patients had to be managed by a hospital hematology department and be between 20 and 80 years old. They had to agree to participate in the study and to be interviewed by a member of the occupational diseases department and they had to understand the French language.

Among these patients, some of them were referred to the occupational diseases consultation by their treating hematologist who suspected an occupational disease. These patients are named “filtered” and the others are named “unfiltered”.

### 2.3. RHELYPRO Questionnaire

The RHELYPRO questionnaire was designed specifically for the study after a review of the literature and discussions with the physicians of the different participating occupational diseases consultation centers. Then, eighteen harmful substances and/or occupational activities associated with a potential increased risk of the occurrence of these hemopathies were selected. This questionnaire is available in Supplementary Materials (Questionnaire S1). It consists of two parts: a “general questionnaire” on patient and disease characteristics and leisure activities and an “occupational” questionnaire designed to define the harmful substances to which the patient had been exposed.

#### 2.3.1. General Questionnaire

The data recorded in the “general questionnaire” were gender, date and place of birth, date of completion of the questionnaire, places and dates of residence of the patient’s entire life, profession of parents and spouse, level of education, existence of training periods, highest diploma obtained, completion of military service and date, tobacco consumption. Patients were asked to give the proximity of their homes to agricultural areas (<50 m or 50–500 m).

Medical data included diagnosis of disease, coded according to the WHO 2016 classification of hematological malignancies [6], date of diagnosis, circumstances of diagnosis, tumor stage according to the modified Ann Arbor classification [7], taking into consideration the extent of the disease and the presence of general clinical signs, lymphoma’s risk factors as history of *Helicobacter Pylori* infection, chronic hepatitis C, constitutional or acquired immune deficiency, systemic lupus erythematosus, celiac disease or Sjogren’s syndrome, family history of blood malignancy in parents and siblings.

Non-occupational exposures to risk factors for the development of blood malignancies were recorded: home close to agricultural areas (distance, type of crop and period); leisure activities such as do-it-yourself, gardening or maintenance of indoor plants with the use of pesticides, insecticides or wood treatment, painting (annual volume of paint used, frequency and duration of use, type and volume of solvents such as white spirit, naphtha solvent, dichloromethane or trichloroethylene) or metal work (type and volume of used solvents, frequency and duration).

#### 2.3.2. Occupational Questionnaire

**Figure 1:**
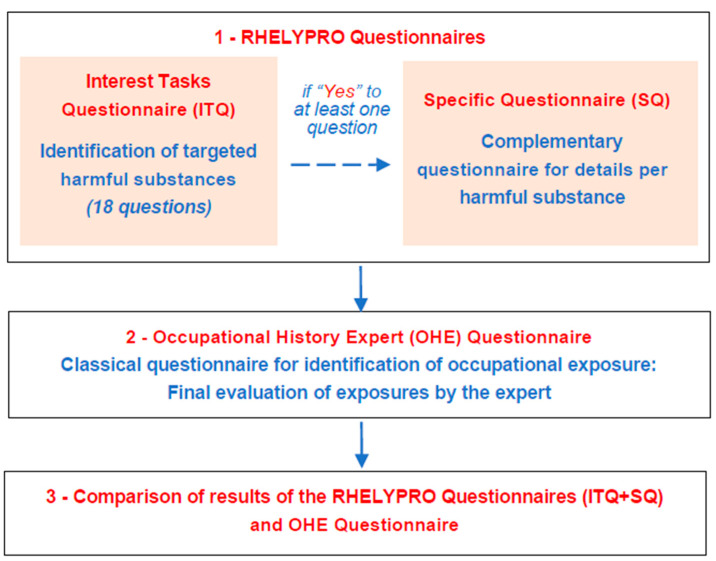
Process for the administration and exploitation of the occupational questionnaires (see in Supplementary Materials: Questionnaire S1 for the Repérage des HEmopathies LYmphoïdes d’origine PROfessionnelle (RHELYPRO) questionnaires and Questionnaire S2 for the Occupational History Expert Questionnaire).

In a first step, each patient completed a multi-part occupational questionnaire summarized in Figure 1 during a face-to-face interview with the investigator. This overall questionnaire consisted of a complete and detailed job history (occupational curriculum), “Task of Interest” questions and then “specific questionnaires” to identify harmful substances in the workplaces (Questionnaire S1). The exposures sought in the questionnaire corresponded to those for which, according to the literature on the subject, there was a potential excess risk of hematological diseases.

- The “Tasks of Interest” part of the questionnaire (ITQ) aimed to identify occupational exposures to harmful substances and/or certain activities via 18 short questions on benzene, ionizing radiation, formaldehyde, pesticides, trichloroethylene, toluene and/or xylene, 1–3 butadiene, pentachlorophenol, ethylene oxide, solvents in general, polychlorinated biphenyls and polychlorinated dibenzodioxins/furans (PCB/PCDD/F). For solvents, a first series of questions specifically explored exposures to trichloroethylene, methylene chloride and gasoline used as a solvent and exposure to other solvents (question 1); a second series explored occupational exposure to toluene and xylene (question 5). It was decided to group in a single category named “various solvents” the answers for toluene, xylene, gasoline as solvent and other solvents identified by question 1. If the patient answered “yes” to at least one questions of the questionnaire, he/she then answered the complementary/specific questionnaire(s) detailed below.
- The other part of the questionnaire, named “Specific Questionnaire”, developed in a collegial manner with all the investigators, was completed each time an exposure of interest was identified in the previous task questionnaire. This specific questionnaire was used to estimate the exposure (probability, frequency, intensity, duration) to each harmful substance of interest.
- The additional form, named “Occupational History Expert (OHE)”, was then completed (Figure 1). This form is routinely used in occupational diseases departments and was considered as the reference questionnaire for the study. It is available as Supplementary Material (Questionnaire S2). The “OHE” questionnaire includes questions relating to each of the tasks performed by the patient in the course of his/her work that may expose him/her to the harmful substances described above. To date, it is on the basis of this questionnaire that the assessment of exposures is carried out by occupational diseases experts in order to determine whether or not a declaration of occupational disease should be made. Professions were coded according to the 2008 International Standard Classification of Occupations (ISCO-08) of the International Labour Organization.

Lastly, the acceptability of the RHELYPRO questionnaire was rated by the patient as easy, moderate or difficult.

### 2.4. Final Evaluation of Exposures by the Expert

During the final evaluation based on the information provided by the “OHE” questionnaire, each physician of the occupational disease departments evaluated the following parameters of exposure to the harmful substances:

- Probability of exposure *P* (1: doubtful, 2: possible, 3: probable, 4: certain)
- Frequency of exposure *F* (1: sporadic, i.e., less than 5% of working time, 2: discontinuous, 3: permanent, i.e., more than 50% of working time)
- Intensity of exposure *I* (1: very low, i.e., close to environmental level, 2: low, i.e., less than 10% of the Occupational Exposure Limit Value 3: medium, 4: high, i.e., above the Occupational Exposure Limit Value) (generally Time Weighted Average, when available).

The intensity of exposure was evaluated by the expert in each center on a semi-quantitative basis according to his/her knowledge of the different occupational situations. Based on evaluation of theses parameters for the different harmful substances over the working-life, each expert could then decide to encourage the patient to claim for recognition of his disease as an occupational disease.

### 2.5. Comparison of the Two Questionnaires

In order to compare the exposures reported in the RHELYPRO ITQ and SQ questionnaires and those retained after the expert’s final estimate from “OHE “, the different questions targeting the same harmful substance were grouped. They are indicated in Table 1 for each of the harmful substances evaluated.

A patient was considered to be exposed to pesticides if he/she answered “yes” to at least one of questions 12 to 17 of the ITQ questionnaire. A patient was considered exposed to benzene if he/she answered “yes” to questions 3 or 4 or reported using benzene as a solvent in question 1. A patient was considered exposed to polychlorinated biphenyls or polychlorinated dibenzodioxins/furans if he/she answered “yes” to questions 10 or 18 of ITQ questionnaire. A patient was considered to be exposed to solvents if he answered “yes” to either question 1 or question 5 of ITQ questionnaire. However, for comparison with the expert assessment, it was also taken into account if he/she had been exposed to trichloroethylene, dichloromethane or other solvents.

### 2.6. Statistical Analyses

Descriptive statistical analyses were performed on all subjects enrolled in the study. Analyses related to occupational exposures were performed in the total population and specifically in filtered or unfiltered patients where appropriate. Indeed, it was considered important to specifically evaluate some parameters in “unfiltered” patients in order to limit selection bias, for evaluating the sensitivity and specificity of the RHELYPRO questionnaire in relation to the expertise.

Qualitative variables were compared using Chi-2 test or Fisher’s exact test. Quantitative variables were compared using Student’s t-test or Mann–Whitney test.

Statistical tests were performed using the website BiostaTGV (https://biostatgv.sentiweb.fr/, accessed on 22 February 2021) based on R Statistical Software. Fisher’s exact test was performed with SAS software v9.4 (SAS Institute Inc., Cary, North Carolina, USA).

The results of the ITQ questionnaires were compared to the results of the final evaluation obtained from the reference questionnaire (“OHE”) by calculating the kappa coefficients of concordance between the two evaluations for each harmful substance. The concordance was considered null if kappa <0, insignificant under 20%, low under 40%, moderate under 60%, good under 80% and very high if 80% or over. The analyses were performed only for occupational exposures and not for other parameters such as non-occupational use of pesticides.

The positive and negative prognostic values of the ITQ questionnaire were calculated. Sensitivity and specificity were calculated when the numbers of patients considered as exposed through the ITQ and OHE questionnaires were greater than 10.

## 3. Results

### 3.1. Patient Disposition and Characteristics

Studies were performed from March 2017 to March 2019 in seven French centers. Information from the RHELYPRO questionnaire and final evaluations were obtained for 210 patients. Five patients were excluded from the study due to inclusion criteria being not met (age over 80 years or diagnosis of blood malignancy made more than 2 years previously; *n* = 4) and memory disorders (*n* = 1). Cooperation was rated as good in 85% of patients.

The patient characteristics are presented in Table 2.

The population of 205 patients was composed of 138 men and 67 women (sex ratio of 2) with a mean age of 59.8 years; 25 patients were “filtered” and 180 “unfiltered”. There were significantly more men than women (*p* = 0.02). Among the lymphoid blood disorders, there were 161 cases of non-Hodgkin’s lymphoma (NHL), (14 T-lymphomas and 147 B-lymphomas), 34 cases of multiple myeloma (MM) and 10 cases of chronic lymphocytic leukemia (CLL). The most common NHL categories according to the World Health Organization classification were diffuse large B-cell lymphomas (*n* = 71), follicular lymphomas (*n* = 41) and mantle cell lymphomas (*n* = 15). These three groups accounted for 79% of the NHL population. The majority of patients had advanced disease: for the 114 patients with known stages of NHL extension, 66 patients were in stage IV, 30 in stage III, 7 in stage II and 11 in stage I.

Fourteen of the 161 NHL patients had a classic risk factor for lymphoma. The most frequent was a family history of blood malignancy (*n* = 10).

Overall, 19% of patients were current smokers, 39% were former smokers and 42% were non-smokers. The mean duration of smoking was 25 years.

The levels of education were distributed as follows: 13% of patients had no diploma, 36% achieved a middle-school degree, 19% had a high-school diploma (baccalaureate) and 27% of patients had a university diploma.

One hundred and twenty (58%) of the 205 patients reported having lived at some point in their lives within 500 m of agricultural areas (32% within 50 m). Concerning their leisure activities, 66 (32%) patients reported painting activities, 6 (3%) metalworking and 38 (19%) pesticide use. Fifty of the 66 patients who had painting activities reported using solvents on these occasions (white spirit, n = 49; trichloroethylene, n = 7 and acetone, n = 2, some patients having used several types of solvents).

Twenty-one percent of the professions were in agriculture and 16% in construction and public works. The professions most involved in situations of highest exposure were farmers, plumbers, printers, machinists and repairers of agricultural machinery.

### 3.2. Evaluation of Exposure to Harmful Substance According to the ITQ Questionnaire

Of the total of 205 patients, 118 (58%) reported occupational exposure: 96 of 180 “unfiltered” patients (53%) and 22 of 25 “filtered” patients (85%). Table 3 describes the details of responses for patients with at least one positive response to the ITQ questionnaire. The “yes” answers most often found for the 96 “unfiltered” patients with at least one positive response to the ITQ questionnaire were question 1 targeting solvents (*n* = 52 patients; 54%), the question targeting gasoline as fuel (*n* = 38; 40%) and the question about working on a farm (*n* = 22; 23%) (Table 3).

The 118 patients with at least one positive response to the ITQ questionnaire were mainly from the agricultural sector and to a lesser extent from the building sector.

### 3.3. Evaluation of Exposure to Harmful Substance According to the Final Evaluation by the Expert

Overall, 111 of the 205 patients had been exposed to at least one harmful substance according to the expert. The four harmful substances most often retained by the expert during the final evaluation were solvents excluding trichloroethylene and dichloromethane, but including toluene and xylene (*n* = 47; 42%), benzene (*n* = 43; 39%), pesticides of any type (*n* = 43, 39%) and trichloroethylene (*n* = 35, 32%).

In “unfiltered” patients, 28 of the 46 (61%) patients identified as exposed to solvents by the ITQ questionnaire were similarly identified by the expert; 28 of the 35 (80%) patients identified as exposed to pesticides by the ITQ questionnaire were similarly identified by the expert (Table 4).

The results of the concordance tests between the ITQ questionnaire and the expert’s assessment for all harmful substances causing hematological malignancies are presented in Table 5.

### 3.4. Diagnostic Value of the RHELYPRO Questionnaire

Among the 205 patients, 49 were identified for at least one exposure using the ITQ questionnaire and 52 by the expert. Results in terms of true positive, false positive, true negative, and false negatives are presented in Table 6 (reference: final evaluation by the expert). Its sensitivity and specificity compared to the final expertise were 96% and 96% for trichloroethylene, 85% and 82% for benzene, 78% and 87% for solvents other than trichloroethylene and dichloromethane, 87% and 95% for pesticides, respectively.

Overall, 41 of the 118 patients (35%) who mentioned an exposure to a hematotoxic substance in the ITQ questionnaire had a proposition for a declaration of occupational disease after evaluation by the expert: 27 “unfiltered” subjects (out of 96; 28%) and 14 “filtered” subjects (out of 22; 64%).

After final expertise, 42 (20%) of the 205 patients were considered to have been exposed to harmful substances potentially responsible for blood malignancies. Of note, 28 (15%) of the 180 “unfiltered” patients benefited from a declaration of occupational disease. One patient had an indirect exposure to trichloroethylene but he did not know he had been exposed to this substance (not screened with the ITQ questionnaire). Among these 28 patients, there was an exposure to pesticides in 18 (64%) patients, to trichloroethylene in 8 (29%), solvents (excluding trichloroethylene and dichloromethane) in 5 (18%) and benzene in 5 (18%).

### 3.5. Evaluation of the RHELYPRO Questionnaire

Out of the total of 205 patients, the questionnaire was judged by the investigator to be easy to use for 162 (79%), not easy for 21 (10.2%) and data were missing for 22 (10.7%). The questionnaire was judged by the patients to be easily acceptable for 136 (66%), moderately acceptable for 25 (12.2%), not easily acceptable for 3 (1.4%) and data were missing for 41 (20%). The mean time spent administering all questionnaires (ITQ and then OHE) was 55 min (range, from 20 to 130 min). If an exposure was detected, the time spent was around 80 min, while it was 50 min when there was none.

## 4. Discussion

Our study showed that the RHELYPRO questionnaire identified the patients exposed to harmful substances associated with the occurrence of lymphoid lymphopathies (49 identified by the questionnaire versus 52 by the expert). This allowed a PPV of 86%, a PNV of 90%, a sensitivity of 92% and a specificity of 83%. Moreover, one third of patients for whom a harmful substance was identified in our RHELYPRO questionnaire were considered by the experts as likely to request recognition as an occupational disease for this type of pathology. In addition, the acceptability of this questionnaire was judged by the patient as acceptable.

Most patients involved in our study (especially those qualified as unfiltered) can be considered representative of patients seen in a standard hematology consultation. Among them, more than half reported an occupational exposure to at least one harmful substance of interest in the RHELYPRO questionnaire, which screened for exposures described associated in the etiological literature. The harmful substances most often reported by these patients were pesticides and solvents (in more than half of the cases).

The relationship between agricultural work, and in particular exposure to pesticides, and NHL was observed by several authors with a dose-relationship. A meta-analysis published in 2019 evaluated the relationship between exposure to pesticides and the risk of developing lymphoid hemopathy in 316,270 farmers. Terbufos and NHL (meta-Hazard Ratio 1.18, 95% CI: 1.00–1.39), deltamethrin and chronic lymphocytic leukaemia/small lymphocytic lymphoma (1.48, 1.06–2.07) and glyphosate and diffuse large B-cell lymphoma (1.36, 1.00–1.85) were moderately but significantly associated [8]. The use of organophosphates and carbamates is associated with a risk of NHL (excluding myeloma) in a study of 1690 cases from 3 U.S. case–control studies and one Canadian study between 1980 and 1990. In this study, there was a positive association between malathion exposure and duration of exposure and the risk of follicular lymphoma [9]. A recent study showed an association between glyphosate and certain forms of NHL, particularly in small cell lymphocytic lymphoma [10].

In our study, several categories of solvents were selected: dichloromethane, trichloroethylene, gasoline fuel and various solvents (gasoline, toluene, xylene and other solvents). However, the relationship between these exposure situations and the occurrence of hematological malignancies has not been firmly established to date. In its evaluation of epidemiological data, the International Agency for Resarch on Cancer (IARC) recognized positive associations between trichloroethylene (TCE) and NHL, with modestly increased risk in the cohort studies and a statistically significant meta-Relative Risk of 1.2 (95% CI: 1.1–1.4) with any exposure to TCE and 1.4 (95% CI: 1.1–1.8) for higher exposure [11]. Dichloromethane, re-evaluated by the IARC in 2017 remains classified in Group 2A (probably carcinogenic to humans), with positive associations between exposure and NHL [12]. Liu et al. observed a pooled Odds Ratio for multiple myeloma of 2.04 (95% CI: 1.31–3.17) in relation to occupational exposure to methylene chloride but not for non-Hodgkin’s lymphoma [13]. Thus, in an American case–control study involving 1189 NHL cases and 982 controls, exposure to chlorinated solvents (1,1,1,-trichloroethane, carbon tetrachloride, chloroform, methylene chloride and perchloroethylene) was evaluated and only an association between carbon tetrachloride exposure and NHL was evidenced [14]. Recent meta-analyses have found no association between benzene exposure and NHL occurrence [15,16]. An absence of relationship was also reported for gasoline emission vapours, at exposure levels encountered in the petroleum industry [17].

A certain number of substances were very rarely detected by the ITQ questionnaire and subsequently by the OHE questionnaire. This was the case for formaldehyde, butadiene, ionizing radiation, ethylene oxide, dichloromethane, polychlorinated dibenzodioxins/furans and pentachlorophenol. Apart from pentachlorophenol [18], the IARC noted that published toxicological studies did not provide any additional evidence for an association between exposure to these substances and the occurrence of NHL. For ethylene oxide, the IARC concluded that it is carcinogenic to humans (Group 1); however, evidence in epidemiological studies was considered limited for the link between NHL and ethylene oxide exposure [19]. For formaldehyde, a meta-analysis published in 2019 did not find an association between occupational exposure to formaldehyde and NHL [20]. In 2009, the IARC considered that numerous studies conducted on butadiene provided strong evidence that the carcinogenicity of that substance involved a genotoxic mechanism, the epidemiological studies from the styrene–butadiene industry showed an excess of leukemia, and a dose–response relationship with cumulative exposure to butadiene, while studies from the monomer industry showed an excess of hematolymphatic malignancies in general, attributable both to leukemia and malignant lymphoma [19]. Since then, in a cohort study of US synthetic rubber workers, the standardized NHL mortality ratio was 1.36 (95% CI: 1.02–1.77), but there was no dose–response relationship for exposure to butadiene and styrene in women [21]. A recent meta-analysis did not confirm a statistical relationship between NHL and occupational PCB exposure (meta-RR 0.94, 95% CI: 0.84–1.03) [22]. The standardized NHL mortality ratio was 1.57 (95% CI: 0.32–4.59) in a New Zealand cohort of 1599 workers between 1969 and 1998 in a production factory of 2,4,5-trichlorophenoxyacetic acid contaminated with 2,3,7,8-tetrachlorodibenzo-*p*-dioxin (TCDD). The authors conclude that these results do not support the carcinogenicity of TCDD at low levels of exposure [23]. The recent meta-analysis of Satta et al. did not show a relationship between exposure to ionizing radiation at dose levels generally encountered in the workplace and the occurrence of lymphomas [24].

For harmful substances with more than 10 positive concordant responses in the ITQ questionnaire and the expert evaluation, agreement can be considered high in “unfiltered” patients for trichloroethylene, strong for pesticides and moderate for solvents and benzene. In this same population, the expert confirmed the exposure reported by the patient in 80% of cases for pesticides and in 60% of cases for solvents. In line with these results, other authors reported previously that agreement between self-reported and expert assessment of exposure was greater for pesticides compared to solvents [25].

The strengths of the RHELYPRO questionnaire are mainly its elaboration by several occupational diseases teams with complementary skills on the different harmful substances of interest involved or suspected to be related to NHL. The RHELYPRO questionnaire was acceptable for a large majority of patients (about 80%). The analysis of the performance of the ITQ questionnaire in relation to the conclusions of the expert shows that it was relevant to identify the harmful substances retained by the expert as probably involved in NHL (kappa coefficient greater than 0.80 for trichloroethylene, pesticides and ionizing radiation). The RHELYPRO ITQ questionnaire appears sensitive and specific. Overall, among all the NHL cases “unfiltered” by the clinicians, 15% of the subjects finally had a proposition for a declaration of occupational disease. Most of the reports concerned exposures to various pesticides (64%), but also exposures to some solvents (trichloroethylene 29%, benzene 18%, solvents other than chlorinated solvents 18%), sometimes in multiple exposures.

This study has some limitations. There were recall difficulties in some patients, for example for dates, quantities and frequencies of use of products handled, places and names of companies and sometimes for a lack of knowledge of the products used. Some patients experienced difficulties in quantifying exposures to harmful substances, particularly for pesticides and solvents, which were the most commonly identified harmful substances. Moreover, the exposure assessment to the different harmful substances was semiquantitative because it was carried out by an occupational expert, and not with any atmospheric metrology. These experts were skilled and participated in training meetings to harmonize their practices. However, the evaluation of the concordance of their conclusions on exposure to harmful substances was not an objective of this study. In future studies, it would be interesting to perform blind comparisons with a predefined number of experts for each harmful substance.

## 5. Conclusions

This study showed that the RHELYPRO questionnaire was a useful tool for identifying occupational exposures to carcinogens in patients with NHL, multiple myeloma or chronic lymphocytic leukemia. Thanks to its acceptability (duration, understandability), this questionnaire allowed the clinician to identify patients for whom a specialized occupational diseases consultation was useful for a more in-depth evaluation of exposures, with a view to a proposal for an occupational disease claim. This questionnaire could also be used in other epidemiological analytical studies on NHL. Moreover, because a large number of harmful substances were covered, parts of this questionnaire might be used for the assessment of occupational exposures in other cancers involving the same carcinogens.

## Figures and Tables

**Table 1:** Correspondence between the harmful substances identified by the expert’s final assessment and “tasks of interest” (ITQ) questions, RHELYPRO Study (2017–2019).

ITQ Questions	Harmful Substance Selected Based on Final Assessment
Q12. Did you work on a farm? Q13. Did you perform any tasks in contact with fruits, vegetables, flowers, for storage, sorting, packing, weighing, selling? Q14. Did you perform tasks in contact with wood (boards, frames, carpentry, furniture, other objects) for example in a logging, sawmill, joinery? Q15. Have you maintained green spaces, roads (including railroad tracks, maintenance of the electrical network, sports fields, public spaces? Q16. Have you disinfected, eliminated rats for reasons of hygiene and public health? Q17. Have you had to treat with pesticides (insecticides, herbicides, fungicides) for any other reason than those just mentioned?	Pesticides without precision and/or atrazine and/or carbamates and/or glyphosate and/or organochlorine insecticides and/or organophosphate insecticides and/or carbaryl and/or carbamates and/or arsenical pesticides and/or toxafen
Q9. Have you been exposed to wood or textiles treatment products?	Pentachlorophenol
Q4. Have you ever had to handle benzene? Q3. Have you ever used gasoline as a fuel or been in contact with gasoline engine fumes? Q1. Have you ever used solvents, degreasers, thinners repeatedly and routinely? Description of using gasoline as a degreaser	Benzene
Use of trichloroethylene detailed in Q1	Trichloroethylene
Use of dichloromethane detailed in Q1	Dichloromethane
Q1. Have you had to use solvents repeatedly and habitually? Q5. Have you used toluene/xylene repeatedly and routinely?	Solvents including toluene and xylene, except trichloroethylene and dichloromethane.
Q2. Have you handled formaldehyde or products containing it?	Formaldehyde
Q6. Have you been exposed to 1,3-butadiene? Q7. Have you been exposed to LPG?	Butadiene
Q10. Have you been exposed to polychlorinated biphenyls (Pyralenes, Arochlors, Phenochlors, Askarels)? Q18. Have you participated in the following tasks/trades/industries (NB: may expose to PCP, PCDD/F, substances not used as such but may be formed in multiple industrial processes)	Polychlorinated biphenyls (PCB), polychlorodibenzodioxins/furans, PCDD/F
Q8. Have you been exposed to ethylene oxide?	Ethylene oxide
Q11. Have you been exposed to sources of ionizing radiation?	Ionizing radiation

LPG: Liquified petroleum gas; PCB, PCDD/F: polychlorinated biphenyls/polychlorinated dibenzodioxins/furans.

**Table 2:** Patient characteristics, RHELYPRO Study (2017–2019).

Characteristics	Total Population (*N* = 205)	“Filtered” Patients (*N* = 25)	“Unfiltered” Patients (*N* = 180)	*p*-Value
Mean age, years	59.8	57.9	60.1	0.13
Male gender, *n* (%)	138 (67.3)	2 (8)	116 (64.4)	0.02
Type of lymphoid blood disorder, *n* (%)				
Non-Hodgkin lymphoma	161 (78.5)	22 (88)	139 (77.2)	0.14
Multiple myeloma	34 (16.6)	1 (4)	33 (18.3)	
Chronic lymphocytic leukemia	10 (4.9)	2 (8)	8 (4.4)	

**Table 3:** Responses to the 18 questions of the ITQ questionnaire in patients with at least one positive response, RHELYPRO Study (2017–2019).

Type of Exposure Detected by ITQ Questionnaire	Responses from the 118 Patients with 1+ Exposure (*N*,%)	Responses from the 96 “Unfiltered” Patients with 1+ Exposure (*N*,%)
Q1. Solvents	69 (58)	52 (54)
Trichloroethylene *	40 (35)	30 (31)
Dichloromethane *	9 (8)	8 (8)
Solvent gasoline and/or “other solvents” *	57 (48)	43 (44)
Q5. Toluene or xylene	11 (9)	8 (8)
“Various solvents” (solvent gasoline and/or “other solvents”) and/or “toluene or xylene”	61 (52)	46 (48)
Q3. Gasoline fuel or engine fumes	51 (43)	38 (40)
Q4. Benzene	18 (15)	15 (16)
Q11. Ionizing Radiation	11 (9)	10 (10)
Q2. Formaldehyde	10 (8)	8 (8)
Q8. Ethylene oxide	3 (2)	3 (3)
Q6. Butadiene	2 (2)	2 (2)
Q7. Liquefied petroleum gas	2 (2)	1 (1)
Q10. Polychlorinated biphenyls	0	0
Questions related to pesticides		
Q12. Working on a farm	31 (26)	22 (23)
Q16. Disinsectisation and rat control work	15 (13)	11 (11)
Q13. Contact with fruits, vegetables, flowers	13 (11)	9 (9)
Q15. Maintenance of green spaces, public spaces	13 (11)	8 (8)
Q9. Wood/textiles treatment products	10 (8)	8 (8)
Q14. Work in contact with wood	10 (8)	4 (4)
Q17. Pesticide handling in another context	5 (4)	4 (4)
Q18. PCB/Polychlorinated Dibenzodioxin Exposure Tasks	4 (3)	3 (3)
Total number of positive responses	278	206

* Several solvents possible per patient.

**Table 4:** Comparisons of the exposures according to ITQ and expert analysis in the “unfiltered” population (*n* = 180). RHELYPRO Study (2017–2019).

Harmful Substance	Patients Exposed According to ITQ	Patients Exposed According to Expert	ITQ+ Expert+	ITQ+ Expert-	ITQ- Expert+	ITQ- Expert-
Solvents including toluene and xylène and excluding TCE and DCM	46	36	28	18	8	126
Benzene	55	33	28	27	5	120
Pesticides ^a^	35	32	28	7	4	141
TCE	30	25	24	6	1	149
Formaldehyde	8	9	6	2	3	169
Radiations	10	7	7	3	0	170
Dichloromethane	8	6	4	4	2	170
Butadiene	2	4	1	1	3	175
Polychlorinated biphenyls/polychlorinated dibenzodioxins/furans	3	3	2	1	1	176
Pentachlorophenol	4	1	0	4	1	175
Ethylene oxide	3	1	1	2	0	177

DCM, dichloromethane; TCE, trichloroethylene; ^a^ multiple possible routes of pesticide exposure (i.e., multiple positive responses to ITQ) for a patient.

**Table 5:** Kappa coefficient between the exposure assessment of each harmful substance according to the ITQ questionnaire and the expert analysis, when more than 5 patients were exposed to (“unfiltered” patients), RHELYPRO Study (2017–2019).

	Kappa
Trichlorethylene	0.83
Ionizing radiation	0.81
Formaldehyde	0.69
Solvents	0.58
Benzene	0.49
Pesticides	0.80
Dichloromethane	0.66

**Table 6:** Diagnostic value of the ITQ questionnaire compared to the expert’s final assessment (*n* = 205 patients). RHELYPRO Study (2017–2019).

	Total	TP	FN	FP	TN	PPV	NPV	Sensitivity	Specificity
Total population	205	102	9	160	78	86%	90%	92%	83%
“Filtered” patients	25	22	1	0	2	100%	99%	96%	100%
“Unfiltered” patients	180	80	8	16	76	83%	90%	91%	83%

TP, true positive; FN, false negative; FP, false positive; TN, true negative; PPV, positive predictive value; PNV, predictive negative value.

## Supplementary Materials

The following are available online at https://www.mdpi.com/article/10.3390/ijerph18084008/s1, Questionnaire S1: RHELYPRO Questionnaires, Questionnaire S2: Occupational History Expert Questionnaire.
