# Supplementary material for: Development of a Questionnaire for the Search for Occupational Causes in Patients with Non-Hodgkin Lymphoma: The RHELYPRO Study
Source: Int J Environ Res Public Health. 2021 Apr 11;18(8):4008. doi: 10.3390/ijerph18084008 (PMC8068898; doi:10.3390/ijerph18084008)
Supplement: Supplementary file 1 [file ijerph-18-04008-s001.zip › ijerph-1140628-FR-supplementary/Questionnaire S1_RHeLyPro Questionnaire_29mar21.docx]

**RHeLyPro**

**Questionnaire**

**GENERAL INFORMATION**

***CHARACTERISTICS OF THE PATIENT AND THE INVESTIGATION***

**Patient Number**: I__I I__I I__I I__I (center, increment number)

**Last name:** I__I **First name:**  I__I *NB: first letter of last name and first name*

**Sex: M**  **F** 

**Date of birth**: __ __ / __ __/__ __ __ __

**Place of birth** (city, department, country if foreign): ……............................................................................................

……………………………………………………………………………………………………………………………………………………………………………………………..

**Referring hematologist:**

Name ……………………………………………………………………… Tel: …………………………….

Address ...................................................................................................................................................................

………………………………………………………………………………………………………………………………………………………………………….

**Attending physician:**

Name ……………………………………………………………………… Tel: …………………………….

Address ...................................................................................................................................................................

………………………………………………………………………………………………………………………………………………………………………….

**Exclusion criteria:**  HIV infection  Previous organ or marrow transplant history

**Inclusion criteria: Diagnosis of**

 NHL  Multiple myeloma  Chronic lymphocytic leukemia

**Diagnosis**:  Incident  Prevalent Date of diagnosis: _ _ / _ _ / _ _ _ _

**If the interview was not performed, specify the reason:**

 Refusal  Lost to follow-up   Death before interview   Impossible communication

**Date of the interview:** __ __ / __ __ /__ __ __ __

**Patient cooperation**: 1: good, 2: fair, 3: poor

**If poor, reason:**

 language problem   confusion / too tired to answer    other, specify:…………………………

**Investigator:** ………………………………………………………………………………………………………………………………………………………………………..

***Residences***

1. **What addresses have you lives at?** (since your birth until today)

| Period 1 | City: ………………………………………… Postal code: |  |  |  |  |  | from |  |  |  |  | to |  |  |  |  |
| --- | --- | --- | --- | --- | --- | --- | --- | --- | --- | --- | --- | --- | --- | --- | --- | --- |

Address: ………………………………………………………………………………………

| Period 2 | City: ………………………………………… Postal code: |  |  |  |  |  | from |  |  |  |  | to |  |  |  |  |
| --- | --- | --- | --- | --- | --- | --- | --- | --- | --- | --- | --- | --- | --- | --- | --- | --- |

Address: ………………………………………………………………………………………

| Period 3 | City: ………………………………………… Postal code: |  |  |  |  |  | from |  |  |  |  | to |  |  |  |  |
| --- | --- | --- | --- | --- | --- | --- | --- | --- | --- | --- | --- | --- | --- | --- | --- | --- |

Address: ………………………………………………………………………………………

| Period 4 | City: ………………………………………… Postal code: |  |  |  |  |  | from |  |  |  |  | to |  |  |  |  |
| --- | --- | --- | --- | --- | --- | --- | --- | --- | --- | --- | --- | --- | --- | --- | --- | --- |

Address: ………………………………………………………………………………………

| Period 5 | City: ………………………………………… Postal code: |  |  |  |  |  | from |  |  |  |  | to |  |  |  |  |
| --- | --- | --- | --- | --- | --- | --- | --- | --- | --- | --- | --- | --- | --- | --- | --- | --- |

Address: ………………………………………………………………………………………

| Period 6 | City: …………………………………………. Postal code: |  |  |  |  |  | from |  |  |  |  | to |  |  |  |  |
| --- | --- | --- | --- | --- | --- | --- | --- | --- | --- | --- | --- | --- | --- | --- | --- | --- |

Address: ………………………………………………………………………………………

| Period 7 | City: ………………………………………… Postal code: |  |  |  |  |  | from |  |  |  |  | to |  |  |  |  |
| --- | --- | --- | --- | --- | --- | --- | --- | --- | --- | --- | --- | --- | --- | --- | --- | --- |

Address: ………………………………………………………………………………………

| Period 8 | City: ………………………………………… Postal code: |  |  |  |  |  | from |  |  |  |  | to |  |  |  |  |
| --- | --- | --- | --- | --- | --- | --- | --- | --- | --- | --- | --- | --- | --- | --- | --- | --- |

Address: ………………………………………………………………………………………

| Period 9 | City: ………………………………………… Postal code: |  |  |  |  |  | from |  |  |  |  | to |  |  |  |  |
| --- | --- | --- | --- | --- | --- | --- | --- | --- | --- | --- | --- | --- | --- | --- | --- | --- |

Address: ………………………………………………………………………………………

| Period 10 | City: ………………………………………… Postal code: |  |  |  |  |  | from |  |  |  |  | to |  |  |  |  |
| --- | --- | --- | --- | --- | --- | --- | --- | --- | --- | --- | --- | --- | --- | --- | --- | --- |

Address: ………………………………………………………………………………………

1. **Have you lived close to agricultural areas (crops, vineyards...)?  Yes  No**

**If yes, please specify the period, the type of crop (vineyards / cereal / vegetables /** **orchards / corn / beets…) and tick the boxes corresponding to the distance and the type of crop.**

| **Period**  **(specify number)** | **Distance** | | | **Type of crop** | |
| --- | --- | --- | --- | --- | --- |
|  | **0 to 50 m** | **50 to 500m** | **>500 m** |  |  |
|  |  |  |  |  |  |
|  |  |  |  |  |  |
|  |  |  |  |  |  |
|  |  |  |  |  |  |
|  |  |  |  |  |  |
|  |  |  |  |  |  |
|  |  |  |  |  |  |
|  |  |  |  |  |  |
|  |  |  |  |  |  |

***JOBS OF RELATIVES***  *NB:* *collect the main occupation during life*

- Father's job: ………………………………………………………………………………………………………………………………………….

- Mother's job: ……………………………………………………………………………………………………………………………………..

- Spouse's job: .............................................................................................................................................

***SCHOOLING AND OCCUPATIONAL TRAINING***

**At what age did you leave general education?**

**What level of education have you completed?**

** **Never attended school ** **Primary

** **Middle School ** **High School ** **

** **Higher education

**Have you completed one or more periods of professional training?  Yes  No**

If yes, please specify, and complete one page of professional activity for each training period.

Training period: Level obtained:

From ---------- to ----------- Level: ……………………………………………………

From ---------- to ----------- Level: …………………………………………………..

From ---------- to ----------- Level: …………………………………………………..

From ---------- to ----------- Level: …………………………………………………..

**Have you completed a period of military activity?  Yes  No**

If yes, specify the period: from -------------- to --------------- *(complete an occupational activity page)*

***SMOKING***

 Non-smoker  Smoker  Ex-smoker (discontinuation for more than one year)

**Duration**: Start Year: __ __ __ __ End year: __ __ __ __

**Total duration of smoking** (in years): __ __ **Cumulative smoking** (pack-years): I__I I__I I__I

*Cf help to calculate consumption expressed in packet-years in leaflet.*

**Other** (cannabis, nargileh, electronic cigarette, etc.) **Specify**(quantity, duration): ……………………………………….

…………………………………………………………………………………………………………………….......................................................

**Have you lived with family members who smoked in your presence?**

** Yes  No** If yes how many people? …………………………

Exposure period: from: ………… to ……….. duration: …………………………..

from: ……...... to ……….. duration: …………………………..

**Have you worked in a place where people smoked in your presence?**

** Yes  No** If yes, how many people? ………………………

Exposure period: from: ………… to ……….. duration: …………………………..

from: ……...... to ……….. duration: …………………………..

**MEDICAL INFORMATION**

*To be completed with the data from the medical file*

**Type of hemopathy according to WHO 2016 classification:** *…………………………………………………………………………………………………………………………………………………….*

*…………………………………………………………………………………………………………………………………………………….*

…………………………………………………………………………………………………………………………………………………….

*NB1: cf leaflet*

*NB2: Attach a copy of the anatomopathology report for NHL.*

1. **Tumor stage (Ann Arbor)**

Known: yes / no

Stage: _ _ /_ (*Cf leaflet)*

1. **Diagnosis date of the disease:**………………………………………………………………………………………

| Date of the anatomopathological results (lymphoma) |  |  | / |  |  | / |  |  |  |  |
| --- | --- | --- | --- | --- | --- | --- | --- | --- | --- | --- |

| Date of bone marrow examination (myeloma) |  |  | / |  |  | / |  |  |  |  |
| --- | --- | --- | --- | --- | --- | --- | --- | --- | --- | --- |

| Date of flow cytometry (chronic lymphocytic leukemia) |  |  | / |  |  | / |  |  |  |  |
| --- | --- | --- | --- | --- | --- | --- | --- | --- | --- | --- |

1. **Non-occupational risk factors:**

| Disorder | No  0 | Yes  1 | DK  2 |
| --- | --- | --- | --- |
| Helicobacter Pylori infection | \|  \| \| --- \| | \|  \| \| --- \| | \|  \| \| --- \| |
| Constitutional immunity deficiency | \|  \| \| --- \| | \|  \| \| --- \| | \|  \| \| --- \| |
| Acquired immune deficiency (long-term corticosteroid therapy,  Immunosuppressive drugs) | \|  \| \| --- \| | \|  \| \| --- \| | \|  \| \| --- \| |
| Family history of malignant hemopathy in father/mother | \|  \| \| --- \| | \|  \| \| --- \| | \|  \| \| --- \| |
| Family history of malignant hemopathy in siblings | \|  \| \| --- \| | \|  \| \| --- \| | \|  \| \| --- \| |
| HCV Infection | \|  \| \| --- \| | \|  \| \| --- \| | \|  \| \| --- \| |
| History of systemic lupus erythematosus | \|  \| \| --- \| | \|  \| \| --- \| | \|  \| \| --- \| |
| History of celiac disease (gluten intolerance proven by gastric fibroscopy or antitransglutaminase antibodies) | \|  \| \| --- \| | \|  \| \| --- \| | \|  \| \| --- \| |
| History of Sjögren's syndrome | \|  \| \| --- \| | \|  \| \| --- \| | \|  \| \| --- \| |

***LEISURE ACTIVITIES***

**Do you practice or have you practiced DIY or gardening as a leisure activity?**

** Yes  No**

**If yes:**

- **Have you had any painting activities?  Yes  No**

How often per year? /_____ / times per year

For how many years: /_____ / years

Volume of paint (in Liters) used per year: /_____ / Liters

During this period, name(s) and volume (in liters) of solvent(s) used per year:

Solvent 1:  White spirit   Naphta  Dichloromethane   Trichlorethylene

Volume/year: /_____ / Liters/year

Solvent 2:  White spirit   Naphta  Dichloromethane   Trichlorethylene Volume/year: /_____ / Liters/year

Solvent 3:  White spirit   Naphta  Dichloromethane   Trichlorethylene Volume/year: /_____ / Liters/year

- **Have you worked with metals?** ** Yes  No**

How often per month: /_____ / times per month

For how many years: /_____ / years

Name(s) and volumes (in Liter) of solvent(s) used per year:

Solvent 1:  White spirit   Naphta   Other, specify _______________________

Volume/year: /_____ / Liters/year

Solvent 2:  White spirit   Naphta   Other, specify _______________________

Volume/year: /_____ / Liters/year

Solvant 3:  White spirit   Naphta   Other, specify _______________________

Volume/year: /_____ / Liters/year

- **Did you use pesticides?**  ** Yes  No**

Notably during gardening activities, treatment of houseplants, treatment against crawling or flying insects, wood treatment)

Name of products used …………………………………………………………………………………………………….

How often per month: /_____ / times per month

For how many years? /_____ / years

Pesticide 1 (specify): _________________________

What volume at each use: /_____ / Liters

What volume per month/year (circle): /_____ / Liters

Pesticide 2 (specify): _________________________

What volume at each use: /_____ / Liters

What volume per month/year (circle): /_____ / Liters

Pesticide 3 (specify): _________________________

What volume at each use: /_____ / Liters

What volume per month/year (circle): /_____ / Liters

**OCCUPATIONAL QUESTIONNAIRE:**

**1a):**

**Complete occupational curriculum**

**Summary of all jobs from the oldest to the most recent, including periods of training, military service and unemployment.**

|  | Start date  End date  Month / year | Sector of activity  of the company  (NAF 2008) | Job  (ISCO 2008) | Social Security System  General: 1 Agriculture: 2  Independent: 3 Civil service: 4 Other: 5 |
| --- | --- | --- | --- | --- |
| **1** | __ __ / __ __ __ __  __ __ / __ __ __ __ | ……………………….................  ……………………………………   \|  \|  \|  \|  \|  \| \| --- \| --- \| --- \| --- \| --- \| | ………..………………...........  …………………………….…….   \|  \|  \|  \|  \|  \| \| --- \| --- \| --- \| --- \| --- \| | ……………...  ………………   \|  \|  \| \| --- \| --- \| |
| **2** | __ __ / __ __ __ __  __ __ / __ __ __ __ | ………………………........................  ……………………………………………..   \|  \|  \|  \|  \|  \| \| --- \| --- \| --- \| --- \| --- \| | ………..………………...........  …………………………….…….   \|  \|  \|  \|  \|  \| \| --- \| --- \| --- \| --- \| --- \| | ……………...  ………………   \|  \|  \| \| --- \| --- \| |
| **3** | __ __ / __ __ __ __  __ __ / __ __ __ __ | ………………………........................  ……………………………………………..   \|  \|  \|  \|  \|  \| \| --- \| --- \| --- \| --- \| --- \| | ………..………………...........  …………………………….…….   \|  \|  \|  \|  \|  \| \| --- \| --- \| --- \| --- \| --- \| | ……………...  ………………   \|  \|  \| \| --- \| --- \| |
| **4** | __ __ / __ __ __ __  __ __ / __ __ __ __ | ………………………........................  ……………………………………………..   \|  \|  \|  \|  \|  \| \| --- \| --- \| --- \| --- \| --- \| | ………..………………...........  …………………………….…….   \|  \|  \|  \|  \|  \| \| --- \| --- \| --- \| --- \| --- \| | ……………...  ………………   \|  \|  \| \| --- \| --- \| |
| **5** | __ __ / __ __ __ __  __ __ / __ __ __ __ | ………………………........................  ……………………………………………..   \|  \|  \|  \|  \|  \| \| --- \| --- \| --- \| --- \| --- \| | ………..………………...........  …………………………….…….   \|  \|  \|  \|  \|  \| \| --- \| --- \| --- \| --- \| --- \| | ……………...  ………………   \|  \|  \| \| --- \| --- \| |
| **6** | __ __ / __ __ __ __  __ __ / __ __ __ __ | ………………………........................  ……………………………………………..   \|  \|  \|  \|  \|  \| \| --- \| --- \| --- \| --- \| --- \| | ………..………………...........  …………………………….…….   \|  \|  \|  \|  \|  \| \| --- \| --- \| --- \| --- \| --- \| | ……………...  ………………   \|  \|  \| \| --- \| --- \| |
| **7** | __ __ / __ __ __ __  __ __ / __ __ __ __ | ………………………........................  ……………………………………………..   \|  \|  \|  \|  \|  \| \| --- \| --- \| --- \| --- \| --- \| | ………..………………...........  …………………………….…….   \|  \|  \|  \|  \|  \| \| --- \| --- \| --- \| --- \| --- \| | ……………...  ………………   \|  \|  \| \| --- \| --- \| |
| **8** | __ __ / __ __ __ __  __ __ / __ __ __ __ | ………………………........................  ……………………………………………..   \|  \|  \|  \|  \|  \| \| --- \| --- \| --- \| --- \| --- \| | ………..………………...........  …………………………….…….   \|  \|  \|  \|  \|  \| \| --- \| --- \| --- \| --- \| --- \| | ……………...  ………………   \|  \|  \| \| --- \| --- \| |
| **9** | __ __ / __ __ __ __  __ __ / __ __ __ __ | ………………………........................  ……………………………………………..   \|  \|  \|  \|  \|  \| \| --- \| --- \| --- \| --- \| --- \| | ………..………………...........  …………………………….…….   \|  \|  \|  \|  \|  \| \| --- \| --- \| --- \| --- \| --- \| | ……………...  ………………   \|  \|  \| \| --- \| --- \| |
| **10** | __ __ / __ __ __ __  __ __ / __ __ __ __ | ………………………........................  ……………………………………………..   \|  \|  \|  \|  \|  \| \| --- \| --- \| --- \| --- \| --- \| | ………..………………...........  …………………………….…….   \|  \|  \|  \|  \|  \| \| --- \| --- \| --- \| --- \| --- \| | ……………...  ………………   \|  \|  \| \| --- \| --- \| |
| **11** | __ __ / __ __ __ __  __ __ / __ __ __ __ | ………………………........................  ……………………………………………..   \|  \|  \|  \|  \|  \| \| --- \| --- \| --- \| --- \| --- \| | ………..………………...........  …………………………….…….   \|  \|  \|  \|  \|  \| \| --- \| --- \| --- \| --- \| --- \| | ……………...  ………………   \|  \|  \| \| --- \| --- \| |
| **12** | __ __ / __ __ __ __  __ __ / __ __ __ __ | ………………………........................  ……………………………………………..   \|  \|  \|  \|  \|  \| \| --- \| --- \| --- \| --- \| --- \| | ………..………………...........  …………………………….…….   \|  \|  \|  \|  \|  \| \| --- \| --- \| --- \| --- \| --- \| | ……………...  ………………   \|  \|  \| \| --- \| --- \| |
| **13** | __ __ / __ __ __ __  __ __ / __ __ __ __ | ………………………........................  ……………………………………………..   \|  \|  \|  \|  \|  \| \| --- \| --- \| --- \| --- \| --- \| | ………..………………...........  …………………………….…….   \|  \|  \|  \|  \|  \| \| --- \| --- \| --- \| --- \| --- \| | .……………...  …………………   \|  \|  \| \| --- \| --- \| |
| **14** | __ __ / __ __ __ __  __ __ / __ __ __ __ | ………………………........................  ……………………………………………..   \|  \|  \|  \|  \|  \| \| --- \| --- \| --- \| --- \| --- \| | ………..………………...........  …………………………….…….   \|  \|  \|  \|  \|  \| \| --- \| --- \| --- \| --- \| --- \| | .……………...  …………………   \|  \|  \| \| --- \| --- \| |
| **15** | __ __ / __ __ __ __  __ __ / __ __ __ __ | ………………………........................  ……………………………………………..   \|  \|  \|  \|  \|  \| \| --- \| --- \| --- \| --- \| --- \| | ………..………………...........  …………………………….…….   \|  \|  \|  \|  \|  \| \| --- \| --- \| --- \| --- \| --- \| | .……………...  …………………   \|  \|  \| \| --- \| --- \| |
| **16** | __ __ / __ __ __ __  __ __ / __ __ __ __ | ………………………........................  ……………………………………………..   \|  \|  \|  \|  \|  \| \| --- \| --- \| --- \| --- \| --- \| | ………..………………...........  …………………………….…….   \|  \|  \|  \|  \|  \| \| --- \| --- \| --- \| --- \| --- \| | .……………...  …………………   \|  \|  \| \| --- \| --- \| |
| **17** | __ __ / __ __ __ __  __ __ / __ __ __ __ | ………………………........................  ……………………………………………..   \|  \|  \|  \|  \|  \| \| --- \| --- \| --- \| --- \| --- \| | ………..………………...........  …………………………….…….   \|  \|  \|  \|  \|  \| \| --- \| --- \| --- \| --- \| --- \| | .……………...  …………………   \|  \|  \| \| --- \| --- \| |
| **18** | __ __ / __ __ __ __  __ __ / __ __ __ __ | ………………………........................  ……………………………………………..   \|  \|  \|  \|  \|  \| \| --- \| --- \| --- \| --- \| --- \| | ………..………………...........  …………………………….…….   \|  \|  \|  \|  \|  \| \| --- \| --- \| --- \| --- \| --- \| | .……………...  …………………   \|  \|  \| \| --- \| --- \| |
| **19** | __ __ / __ __ __ __  __ __ / __ __ __ __ | ………………………........................  ……………………………………………..   \|  \|  \|  \|  \|  \| \| --- \| --- \| --- \| --- \| --- \| | ………..………………...........  …………………………….…….   \|  \|  \|  \|  \|  \| \| --- \| --- \| --- \| --- \| --- \| | .……………...  …………………   \|  \|  \| \| --- \| --- \| |
| **20** | __ __ / __ __ __ __  __ __ / __ __ __ __ | ………………………........................  ……………………………………………..   \|  \|  \|  \|  \|  \| \| --- \| --- \| --- \| --- \| --- \| | ………..………………...........  …………………………….…….   \|  \|  \|  \|  \|  \| \| --- \| --- \| --- \| --- \| --- \| | .……………...  …………………   \|  \|  \| \| --- \| --- \| |

**Interest Tasks Questionnaire**

**(ITQ)**

**OCCUPATIONAL QUESTIONNAIRE:**

**1b:**

**Additional questions on harmful substances**

1. **In the course of your career, have you had to repeatedly and habitually use solvents, degreasers (for metals), thinners (in glues, paints etc.)?**

** Yes  No**

**If yes, can you specify which ones, and if so, specify the job number:**

** Trichloroethylene** employment (s) n: ………………………………..

Notably: Electrician/Electronic Engineer, Auto repair/Machine Mechanics, Metallurgy, Synthesis Activities, Packaging, Transportation of Trichloroethylene, Dry Cleaning

*Cf. questionnaire 6 part 1c*

** Methylene Chloride (Dichloromethane)** employment (s) n: ……………………..

Notably: Chemical analysis laboratories, Printed circuit board manufacturing, Leather industry, Cellulosic fiber manufacturing, Cosmetic aerosol production, Use as extraction and process solvent in the food industry, Paint stripping of facades, wall paintings, frames, shutters etc., Textile stain remover manufacturing, Auto repair/machine mechanics, Metallurgy, Rubber/plastics industry, Wood processing and woodworking.

*Cf. questionnaire 7 part 1c*

** Gasoline, used as a degreaser** employment(s) n: ………………………………..

*Cf. questionnaire 3 part 1c*

** Other solvents, specify their name(s)** ……………………………………………………………..

** **employment(s) n: …………………………….

If yes, specify the sources of exposure: …………………………………………………………………………………………

During which cumulative period (years) I__I** **I__I

How often? number of days per month: I__I** **I__I** days/month ;**

Number of hours per day: I__I** **I__I** hours/day**

What volume (in liters) did you use daily/weekly? I__I I__I I__I

*NB:* *Delete as appropriate*

*Remember to detail in the description of the corresponding workstation the duration of use of each of the solvents, the frequency of use (per month), the volume used (in liters per month), as well as the wearing of personal protective equipment when using these products.*

1. **During your career, have you handled formaldehyde or products containing it?  Yes  No  DK**

*Cf. questionnaire 4 part 1c*

Notably: Thanatopraxy, Use as a preservative, Formaldehyde synthesis, Disinfection operations, Medical analysis or pathology laboratory, Sterilization of eggs, Manufacturing of disinfectant products containing formaldehyde, Varnishing and vitrification of floors and furniture, Smoking of meat and fish, Textile industry, Metallurgy, Particleboard, Medium density fibers, plywood, frames, glued laminated timber.

If yes, specify the sources of the exposure: …………………………………………………………………………………………

…………………………………………………………………………………………………………………………………………………………….

During which cumulative period (years) I__I** **I__I

How often? Number of days per month: I__I** **I__I** days/month;**

Number of hours per day: I__I** **I__I** hours/day**

What volume (in liters) did you use daily/weekly?** ** I__I I__I I__I

*NB: Delete as appropriate*

If exposure to formaldehyde results from the use of particleboard etc: specify how many hours per day the panels were handled: hours/day: **  **hours/day.

*Remember to detail in the description of the corresponding workstation the duration of use of formaldehyde or products containing it, the frequency of use (per month), the volume used (in liters per month), as well as the wearing of personal protective equipment when using these products.*

1. **During your career, have you ever used gasoline as a fuel or come into contact with gasoline engine fumes?**

*Cf. questionnaire 3 part 1c*

** Yes  No  DK**

Notably: workstation exposed to gasoline engine fumes from vehicular traffic or gasoline-powered machinery (e.g., agricultural machinery, lawn mowers).

1. **During your career, have you been required to use benzene?**

*Cf. questionnaire 3 part 1c*

** Yes  No  DK**

If yes, specify the sources of the exposure: …………………………………………………………………………………………

During which cumulative period (years)? I__I** **I__I

Frequency of use? Number of days per month: I__I** **I__I** days/months;**

Number of hours per day: I__I** **I__I** hours/day**

What volume (in liters) did you use daily/weekly?** ** I__I I__I I__I

*NB: delete as appropriate*

Notably: Research Laboratory, Physical/Natural Sciences Analysis, Education, Rubber Dissolving Products, Petroleum Refinery, Manufacture of Synthetic Intermediate Chemicals or Perfumes, Printing, Production of Varnish/ Paints/Enamels/Mastics/Glues/Maintenance Products, Metallurgy, Auto repair/Mechanics, Electrician/Electronic Engineer, Production of Varnish/ Paints, Degreasing Operations in the Textile Industry, Chemical or Pharmaceutical Industry

1. **In the course of your career, have you used toluene, xylene, etc. ?**

** Yes  No  DK**

If yes, specify the sources of the exposure: …………………………………………………………………………………………

During which cumulative period (years) I__I** **I__I

Frequency of use? Number of days per month: I__I** **I__I** days/months;**

Number of hours per day: I__I** **I__I** hours/day**

What volume (in liters) did you use daily/weekly?** ** I__I I__I I__I

*NB: delete as appropriate*

1. **During your career, have you been exposed to 1,3-butadiene?**

** Yes  No  DK**

*Cf. questionnaire 9 part 1c*

Notably: Butadiene synthesis, packaging, transportation, rubber/plastics sector

If yes, specify the sources of the exposure: …………………………………………………………………………………………

During which cumulative period (years) I__I** **I__I

Frequency of use? Number of days per month: I__I** **I__I** days/months;**

Number of hours per day: I__I** **I__I** hours/day**

What volume (in liters) did you use daily/weekly?** ** I__I I__I I__I

*NB: delete as appropriate*

1. **In the course of this job, were you exposed to**  liquified petroleum gas**?**

** Yes  No  DK**

*Cf. questionnaire 9 part 1c*

1. **During your career, have you been exposed to ethylene oxide?**

** Yes  No  DK**

*Cf. questionnaire 10 part 1c*

Notably: Activities of synthesis, packaging, transport of ethylene oxide, Sterilization operations or use of medical equipment, Manufacture of ethanolamines, non-ionic surfactants or ethylene glycol

If yes, specify the sources of the exposure: …………………………………………………………………………………………

During which cumulative period (years) I__I** **I__I

Frequency of use? Number of days per month: I__I** **I__I** days/months;**

Number of hours per day: I__I** **I__I** hours/day**

What volume (in liters) did you use daily/weekly?** ** I__I I__I I__I

*NB: delete as appropriate*

1. **During your career, have you been exposed to wood treatment products or textiles?**

** Yes  No  DK**

*Cf. questionnaire 5 part 1c*

Notably: Textile industry, Chipboard/medium density fiberboard/ plywood/ structural work/ glue-laminated timber, Processing of wooden elements/woodworking, Furniture.

If so, do you know if these wood treatment products contained:

Creosote ** Yes  No  DK**

Copper-chromium-arsenic ** Yes  No  DK**

Polychlorophenol ** Yes  No  DK**

Another substance ** Yes.** In this case, specify: …………………………………………………….

Specify the sources of the exposure: …………………………………………………………………………………………

During which cumulative period (years) I__I** **I__I

Frequency of use? Number of days per month: I__I** **I__I** days/months;**

Number of hours per day: I__I** **I__I** hours/day**

What volume (in liters) did you use daily/weekly?** ** I__I I__I I__I

*NB: delete as appropriate*

1. **During your career, have you been exposed to polychlorinated biphenyls (PCBs, Pyralenes, Arochlors, Phenochlors, Askarels...)?**

** Yes  No  DK**

*Cf. questionnaire 8 part 1c*

Notably: Activities of synthesis, packaging, transport of PCBs, linoleum contact.

If yes, specify the sources of the exposure: …………………………………………………………………………………………

During which cumulative period (years) I__I** **I__I

Frequency of use? Number of days per month: I__I** **I__I** days/months;**

Number of hours per day: I__I** **I__I** hours/day**

What volume (in liters) did you use daily/weekly?** ** I__I I__I I__I

*NB: delete as appropriate*

1. **During your career, have you been exposed to sources of ionizing radiations?**

** Yes  No  DK**

*Cf. questionnaire 11 part 1c*

Notably: Airport surveillance, Use of devices containing radioactive sources (humidifiers, gamma-densitometers, radiometric thickness or level gauges, ionizers, ion smoke detectors, detection of the presence of lead), Sterilization or disinfection operations, Air atomic testing, Nuclear industry/Uranium ores, Industry or laboratory, Medical imaging department, interventional cardiology, operating room or equivalent.

If yes, specify the sources of the exposure: ………………………………………………………………………………………

…………………………………………………………………………………………………………………………………………………………….

During which cumulative period (years) I__I** **I__I

Frequency of use? Number of days per month: I__I** **I__I** days/months;**

Number of hours per day: I__I** **I__I** hours/day**

If yes, did you have a dosimeter? ** Yes  No**

**Do you have the dosimetry results?:  Yes  No - Specifiy: …………………………………………..**

1. **In your occupational life, have you ever worked on a farm?**

Including wood industry, seasonal tasks, miscellaneous work (cleaning, maintenance,...) on a farm.

** Yes  No**

*If yes, questionnaire 1 part 1c*

1. **In your occupational life, have you ever worked in contact with fruit, vegetables, flowers, for storage, sorting, packaging, weighing, sales?** ** Yes  No**

*If yes, question 2.1 part 1c*

1. **In your occupational life, have you ever worked with wood (boards, carpentry, joinery, furniture, other objects) e.g in a logging operation, sawmill, carpentry?**

** Yes  No**

*If yes, question 2.2 part 1c*

1. **In your occupational life, have you ever maintained green spaces, roadways (including railroad tracks, power grid maintenance, sports fields, public spaces (parks, cemeteries, sidewalks, roadsides, etc.)?**

** Yes  No**

*If yes, question 2.3 part 1c*

1. **In your occupational life, have you ever killed pests, or rats, for reasons of hygiene and public health (storage, commercial, housing premises)?**

** Yes  No**

*If yes, question 2.4 part 1c*

1. **In your occupational life, have you ever applied pesticides (insecticides, herbicides, fungicides) for any reason other than those previously mentioned?**

** Yes  No**

*If yes, question 2.5 part 1c*

1. **Have you participated in the following tasks/trades/industries (NB: tasks, sectors or substances that may expose to PCBs, PCD, PCDD/F, substances not used as such but that may be formed in multiple industrial processes) ?**

** Yes  No**

*If yes, questionnaire 8 part 1c*

Woodworking, Rubber/Plastics Industry, Electrician/Electronic Engineer, Manufacturing of Herbicides or Wood Treatment Products, Production of Chlorobenzenes/Chlorophenols, Chlorinated Dioxazine Derivative Dyes, Paper Manufacturing Sector, Particleboard, Paints, Glues, Inks, Food Industry/Fuel Vessels, Industrial or Domestic Incinerators, Cement Plant Chimney Maintenance

**Specific Questionnaire**

**(SQ)**

**OCCUPATIONAL QUESTIONNAIRE**

**1c:**

**Specific harmful substances questionnaires**

1. **Agricultural pesticides on farms (livestock and crops)……………..……................. pages 18-21**
2. **Non-agricultural pesticides …………………………………………………………………………… pages 22-23**
3. **Benzene…………………………………………………………………………………………………………. pages 24-30**
4. **Formaldehyde………………………………………………………………………………………………… pages 31-34**
5. **Pentachlorophenol…………………………………………………………………………………………. page 35**
6. **Trichlorethylene…………………………………………………………………………………………….. pages 36-37**
7. **Dichloromethane……………………………………………………………………………………………. pages 38-41**
8. **PCB/PCDD………………………………………………………………………………………………………. pages 42-45**
9. **Butadiene ……………………………………..………………………………………………………………. pages 46-47**
10. **Ethylene oxide ……………………………..……………………………………………………………….. page 48**
11. **Ionizing radiation …………………………………………………………………………………………… pages 49-51**
    1. **Agricultural pesticide questionnaire**

- **Have you ever carried out activities related to one (or more) farm(s)?**  YES I_I NO I_I

- **On these farms**

Have you participated in harvesting activities? (grape harvest/hay) YES I_I NO I_I

Did you take care of the green spaces? YES I_I NO I_I

Have you weeded the courtyard, the embankments, the alleys? YES I_I NO I_I

Did you live on the farm? YES I_I NO I_I

- **Have you performed any activities related to a livestock operations?** YES I_I NO I_I

| **Farm 1**  Start Year: …………... End Year: ……………  Name and address    Surface area of the farm…………………………………………………  Job position …………………………………………………………………  Status: holder I_I; employee I_I; family helper I_I; other I_I  Crop/livestock treatment? YES I_I NO I_I  Other tasks: |  | **Farm 2**  Start Year: …………... End Year: ……………  Name and address    Surface area of the farm…………………………………………………  Job position …………………………………………………………………  Status: holder I_I; employee I_I; family helper I_I; other I_I  Crop/livestock treatment? YES I_I NO I_I  Other tasks: |  | **Farm 3**  Start Year: …………... End Year: ……………  Name and address    Surface area of the farm…………………………………………………  Job position …………………………………………………………………  Status: holder I_I; employee I_I; family helper I_I; other I_I  Crop/livestock treatment? YES I_I NO I_I  Other tasks: |
| --- | --- | --- | --- | --- |
|  |  |  |  |  |
| **Farm 4**  Start Year: …………... End Year: ……………  Name and address    Surface area of the farm…………………………………………………  Job position …………………………………………………………………  Status: holder I_I; employee I_I; family helper I_I; other I_I  Crop/livestock treatment? YES I_I NO I_I  Other tasks: |  | **Farm 5**  Start Year: …………... End Year: ……………  Name and address    Surface area of the farm…………………………………………………  Job position …………………………………………………………………  Status: holder I_I; employee I_I; family helper I_I; other I_I  Crop/livestock treatment? YES I_I NO I_I  Other tasks: |  | **Farm 6**  Start Year : …………... End Year: ……………  Name and address    Surface area of the farm…………………………………………………  Job position …………………………………………………………………  Status: holder I_I; employee I_I; family helper I_I; other I_I  Crop/livestock treatment? YES I_I NO I_I  Other tasks: |

| **Cattle**  YES I_I NO I_I  Start Year: …………... End Year: ……………  Mean number: ………….. animals  Type of breeding: Milk I_I ; Meat I_I    Use of insecticides on animals? YES I_I; NO I_I  If Yes: Start Year: …………. End Year: …………..  Did you treat livestock buildings? YES I_I; NO I_I  If yes: Start Year:…………. End Year: ………….. |  | **Sheep/ewes/goats**  YES I_I NO I_  Start Year: …………... End Year: ……………  Mean number: ………….. animals  Type of breeding: Milk I_I ; Meat I_I    Use of insecticides on animals? YES I_I; NO I_I  If Yes: Start Year: …………. End Year: …………..  Did you treat livestock buildings? YES I_I; NO I_I  If yes: Start Year:…………. End Year: ………….. |  |  | **Horses**  YES I_I NO I_  Start Year: …………... End Year: ……………  Mean number: ………….. animals    Use of insecticides on animals? YES I_I; NO I_I  If Yes: Start Year: …………. End Year: …………..  Did you treat livestock buildings? YES I_I; NO I_I  If yes: Start Year:…………. End Year: ………….. |
| --- | --- | --- | --- | --- | --- |
|  |  |  |  |  |  |
| **Pigs**  YES I_I NO I_  Start Year: …………... End Year: ……………  Mean number: ………….. animals    Use of insecticides on animals? YES I_I; NO I_I  If Yes: Start Year: …………. End Year: …………..  Did you treat livestock buildings? YES I_I; NO I_I  If yes: Start Year:…………. End Year: ………….. |  | **Poultry**  YES I_I NO I_  Start Year: …………... End Year: ……………  Mean number: ………….. animals  Type of poultry: …………………………………………………..    Use of insecticides on animals? YES I_I; NO I_I  If Yes: Start Year: …………. End Year: …………..  Did you treat livestock buildings? YES I_I; NO I_I  If yes: Start Year:…………. End Year: ………….. |  |  | **Others**  YES I_I NO I_  Start Year: …………... End Year: ……………  Mean number: ………….. animals  Type of animals: …………………………………………………..  Use of insecticides on animals? YES I_I; NO I_I  If Yes: Start Year: …………. End Year: …………..  Did you treat livestock buildings? YES I_I; NO I_I  If yes: Start Year:…………. End Year: ………….. |

- **If you have applied insecticide treatments on animals:**

How: By dipping animals in insecticide baths ** **YES ** **NO

By spraying pesticides with a lance or manual sprayer ** **YES ** **NO

By external application with a pour-on system ** **YES ** **NO

Other: ****………………………………………………………………………………………………………………………………….

- **If you have treated livestock buildings with insecticides:**

How: Insecticide paint ** **YES ** **NO

Spraying on surfaces ** **YES ** **NO

Fumigation ** **YES ** **NO

Other: ****………………………………………………………………………………………………………………………………….

- **Have you treated the wood for gates/fences/posts for enclosures or buildings?  **YES ** **NO

Do you remember the name(s) of the product(s) used on the wood?.................................................................................................

- **Did you perform activities related to a crop(s)? YES I_I NO I_I**

| **Corn**  **YES I_I NO I_I**  Start Year: …………... End Year: ……………  Approximate surface area: ………….. hectares  Type de Corn: Grain I_I ; Fodder I_I ; Seeds I_I    Did you apply pesticides? YES I_I  NO I_I  If yes: Start Year:…………. End Year: …………..  Did you treat the seeds yourself? YES I_I  NO I_I  If yes: Start Year:…………. End Year: ………….. |  | **Wheat/Barley/Cereals YES I_I NO I_I**  Start Year: …………... End Year: ……………  Approximate surface area: ………….. hectares  Type of Cereals: ………………………………………………..    Did you apply pesticides? YES I_I  NO I_I  If yes: Start Year:…………. End Year: …………..  Did you treat the seeds yourself? YES I_I  NO I_I  If yes: Start Year:…………. End Year: ………….. |  | **Rape**  **YES I_I NO I_I**  Start Year: …………... End Year: ……………  Approximate surface area: ………….. hectares    Did you apply pesticides? YES I_I  NO I_I  If yes: Start Year:…………. End Year: …………..  Did you treat the seeds yourself? YES I_I  NO I_I  If yes: Start Year:…………. End Year: ………….. |
| --- | --- | --- | --- | --- |
|  |  |  |  |  |
| **Sunflower YES I_I NO I_I**  Start Year: …………... End Year: ……………  Approximate surface area: ………….. hectares    Did you apply pesticides? YES I_I  NO I_I  If yes: Start Year:…………. End Year: …………..  Did you treat the seeds yourself? YES I_I  NO I_I  If yes: Start Year:…………. End Year: ………….. |  | **Tobacco**  **YES I_I NO I_I**  Start Year: …………... End Year: ……………  Approximate surface area: ………….. hectares    Did you apply pesticides? YES I_I  NO I_I  If yes: Start Year:…………. End Year: …………..  Did you treat the seeds yourself? YES I_I  NO I_I  If yes: Start Year:…………. End Year: ………….. |  | **Meadows**  **YES I_I NO I_I**  Start Year: …………... End Year: ……………  Approximate surface area: ………….. hectares  Type de Prairies: ………………………………………………..    Did you do apply pesticides? YES I_I  NO I_I  If yes: Start Year:…………. End Year: ………….. |
|  |  |  |  |  |
| **Potatoes YES I_I NO I_I**  Start Year: …………... End Year: ……………  Approximate surface area: ………….. hectares    Did you apply pesticides? YES I_I  NO I_I  If yes: Start Year:…………. End Year: …………..  Did you treat the seeds yourself? YES I_I  NO I_I  If yes: Start Year:…………. End Year: ………….. |  | **Beets YES I_I NO I_I**  Start Year: …………... End Year: ……………  Approximate surface area: ………….. hectares  Type of beets: Sugar I_I ; Fodder I_I ;    Did you apply pesticides? YES I_I  NO I_I  If yes: Start Year:…………. End Year: …………..  Did you treat the seeds yourself? YES I_I  NO I_I  If yes: Start Year:…………. End Year: ………….. |  | **Vegetables YES I_I NO I_I**  Start Year: …………... End Year: ……………  Approximate surface area: ………….. hectares  Type of vegetables: …………………………………………………  Under greenhouses I_I ; Open field I_I ;    Did you apply pesticides? YES I_I  NO I_I  If yes: Start Year:…………. End Year: ………….. |
|  |  |  |  |  |
| **Vineyards YES I_I NO I_I**  Start Year: …………... End Year: ……………  Approximate surface area: ………….. hectares  Space between rows: ……………………………………..  Did you apply pesticides? YES I_I  NO I_I  If yes: Start Year:…………. End Year: …………..  Other tasks in contact with the vineyards: …………………………………………………………………………………………  …………………………………………………………………………………………  ……………………………………………………………………………… |  | **Fruit trees YES I_I NO I_I**  Start Year: …………... End Year: ……………  Approximate surface area: ………….. hectares  Type of fruits:…………………………………………………………  Number of trees/ha: …………………………………………..  Did you apply pesticides? YES I_I  NO I_I  If yes: Start Year:…………. End Year: ………….. Other tasks in contact with trees: ………………………………………………………………………………  ………………………………………………………………………………………… |  | **Others YES I_I NO I_I**  Start Year: …………... End Year: ……………  Approximate surface area: ………….. hectares    Did you do apply pesticides? YES I_I  NO I_I  If yes: Start Year:…………. End Year: ………….. |

- **If you have applied pesticides on crops(including chemical weeding)**
- **How many treatment days per year in average:** ………………….. days
- **Were you personally in charge:**
  - of the preparation of the mixtures? YES I_I NO I_I If yes, number of preparations per day:……….
  - of the application of the mixture? YES I_I NO I_I
  - of the cleaning of equipment? YES I_I NO I_I If yes, number of cleanings per year:…………..
  - of the repairs of the equipment YES I_I NO I_I if yeas, number of days per year:……………………..
- **Which equipment did you use for the treatments?**
  - An inter-rank tractor YES I_I NO I_I If yes, did it have a closed cab? YES I_I NO I_I
  - A straddle tractor YES I_I NO I_I If yes, did it have a closed cab? YES I_I NO I_I
  - A self-propelled tractor YES I_I NO I_I If yes, did it have a closed cab? YES I_I NO I_I
  - Another type of tractor:………………………………… If yes, did it have a closed cab? YES I_I NO I_I
  - A trailed sprayer YES I_I NO I_I If yes, what was the volume of the tank …………..Liters
  - A mounted sprayer YES I_I NO I_I If yes, what was the volume of the tank ………….Liters
  - A knapsack sprayer YES I_I NO I_I If yes, number of days per year ………………
- **Were you wearing gloves**
  - During the preparation? Never I_I Sometimes I_I Often I_I Always I_I
  - During the application? Never I_I Sometimes I_I Often I_I Always I_I
  - During the cleaning? Never I_I Sometimes I_I Often I_I Always I_I

Type of gloves:…………………………………………………………………………………………………………………………………………………

- **Were you wearing a coverall?**
  - During the preparation? Never I_I Sometimes I_I Often I_I Always I_I
  - During the application? Never I_I Sometimes I_I Often I_I Always I_I
  - During the cleaning? Never I_I Sometimes I_I Often I_I Always I_I

Type of wetsuit:…………………………………………………………………………………………………………………………………….

- **Were you wearing a mask**
  - During the preparation? Never I_I Sometimes I_I Often I_I Always I_I
  - During the application? Never I_I Sometimes I_I Often I_I Always I_I
  - During the cleaning? Never I_I Sometimes I_I Often I_I Always I_I

Type of mask:…………………………………………………………………………………………………………………………………………………

**Collect from the patient any lists, invoices or treatment schedules that include names of pesticide products or active ingredients.**

- 1. **Non-agricultural pesticide questionnaire**
  2. **In one or several of your jobs, have you ever performed tasks in contact with fruits, vegetables, flowers for storage, sorting, packaging, weighing, selling....?**

 Yes  No

Job Numbers: I_I I_I I_I I_I I_I I_I I_I I_I I_I I_I I_I I_I I_I I_I

Year of start of these tasks: _ _ _ _ Year of end: _ _ _ _

Which plant was it exactly?......................................................................................

What were the tasks performed? …………………………………………………………………………………………….

Have you personally been in charge of treating them with pesticides? YES I_I NO I_I

How many days a year have you applied pesticides?: I_I I_I I_I

Which equipment did you use for these treatments?………………………………………………………………….

Were you wearing protective equipment?: never I_I sometimes I_I often I_I always I_I

Which ones: ……………………………………………………………………………………………………………….

Do you remember which products were used? YES I_I NO I_I - Specify:…………………………………………………………………………………………………………………………………….

- 1. **In one or several of your jobs, did you perform tasks in contact with wood (boards, carpentry, joinery, furniture, other objects) e.g. in a logging operation, sawmill, joinery?**

 Yes  No

Job Numbers: I_I I_I I_I I_I I_I I_I I_I I_I I_I I_I I_I I_I I_I I_I

Year of start of these tasks: _ _ _ _ Year of end: _ _ _ _

What kind of wooden object was it exactly?...............................................................

What were the tasks performed? …………………………………………………………………………………………….

Have you personally been in charge of treating them with pesticides? YES I_I NO I_I

How many days a year did you do these treatments?: I_I I_I I_I

Which equipment did you use for these treatments?………………………………………………………………….

Were you wearing protective equipment?: never I_I sometimes I_I often I_I always I_I

Which ones: ……………………………………………………………………………………………………………….

Do you remember which products were used? YES I_I NO I_I - Specify:…………………………………………………………………………………………………………………………………….

- 1. **In one or several of your jobs, have you maintained green spaces, roads, sports fields, public spaces (parks, cemeteries, sidewalks, roadsides) ...?**

 Yes  No

Job Numbers: I_I I_I I_I I_I I_I I_I I_I I_I I_I I_I I_I I_I I_I I_I

Year of start of these tasks: _ _ _ _ Year of end: _ _ _ _

What type of green space was it exactly?...............................................................

What were the tasks performed? …………………………………………………………………………………………….

Have you personally been in charge of treating them with pesticides? YES I_I NO I_I

How many days a year did you do these treatments?: I_I I_I I_I

Which equipment did you use for these treatments?………………………………………………………………….

Were you wearing protective equipment?: never I_I sometimes I_I often I_I always I_I

Which ones: ……………………………………………………………………………………………………………….

Do you remember which products were used? YES I_I NO I_I Specify:…………………………………………………………………………………………………………………………………….

- 1. **In one or several of your jobs, have you killed pests or mouse/rats for reasons of hygiene and public health (storage rooms, commercial premises, housing, etc.)?**

 Yes  No

Job Numbers: I_I I_I I_I I_I I_I I_I I_I I_I I_I I_I I_I I_I I_I I_I

Year of start of these tasks: _ _ _ _ Year of end: _ _ _ _

In which place(s) exactly did you carry out these treatments?.......................................

Have you personally been in charge of treating them with pesticides? YES I_I NO I_I

How many days a year did you do these treatments?: I_I I_I I_I

Which equipment did you use for these treatments?………………………………………………………………….

Were you wearing protective equipment?: never I_I sometimes I_I often I_I always I_I

- 1. **In one or several of your jobs, have you ever used pesticides for any other reason?**

 Yes  No

Job Numbers: I_I I_I I_I I_I I_I I_I I_I I_I I_I I_I I_I I_I I_I I_I

Year of start of these tasks: _ _ _ _ Year of end: _ _ _ _

Why exactly did you carry out these treatments?.......................................

Have you personally been in charge of treating them with pesticides? YES I_I NO I_I

How many days a year did you do these treatments?: I_I I_I I_I

What equipment did you use for these treatments?………………………………………………………………….

Were you wearing protective equipment?: never I_I sometimes I_I often I_I always I_I

Which ones: ………………………………………………………………………………………………………………

Do you remember which products were used? YES I_I NO I_I Specify:…………………………………………………………………………………………………………………………………..

- 1. **Benzene questionnaire**

## Did you come into contact with petrol fuel during preparation, transfer, handling or tank work?

 Yes  No

Specify: frequency of the task (per month), quantities handled (liters), protective equipment used.

………………………………………………………………………………………………………………………………………………….

## Have you worked as a tanker truck driver?  Yes  No

Specify the number of daily deliveries: I_I I_I I_I

For each delivery, specify the duration of exposure due to tanks loaded/unloaded from the top/bottom: I_II_II_I hours/minutes (Delete as appropriate)

Did you wear respiratory protection during these loading/unloading operations, and if so, what type?  Yes  No

……………………………………………………………………………………………………………………………………………………………..

## As a pump attendant, how many vehicles did you fill each day? I_II_I II_I

## As a fuel pump repairer:

How many interventions did you do per day? I_II_I I_I

What was the proportion of gasoline tanks?......................................................................................

## If you were a service station manager, how many fuel deliveries did you supervise each year? I_II_I I_I

*Exposure to benzene vapors when checking the depletion level and then repletion of the tank* ……………………………………………………………………………………………………………………………………………….

## Have you been exposed to fumes from gasoline engines as a result of vehicular traffic (toll booth, traffic warden, delivery driver, city bus driver, cabs)?

*NB: relatively low exposure to benzene* ** Yes  No**

During which type(s) of ctivity(ies)? ........................................................................................................

……………………………………………………………………………………………………………………………………………………………..

## If you were travelling, specify the number of kilometers travelled per year in an urban area. I_II_II_II_I km/year

## If you were a traffic officer, specify the time spent per week/month in an urban area with high traffic density: NB: relatively low exposure to benzene

I_II_II_I hours per week /month (circle)

## If you were a toll collector, was the road heavily trafficked  Yes  No

Specifiy: …………………………………………………………………………………………………………………………………………….

## Did you drive gasoline-powered farm equipment? NB: Exposure to benzene

** Yes  No**

How many hours per day? I_II_I hours/days

How many days per year? I_II_II_I days/year

## Have you been exposed in close proximity (less than 5 meters) to exhaust fumes from gasoline-powered machines (such as thermal lawnmowers or leaf blowers)?

** ** *NB: Exposure to benzene***  Yes  No**

If yes, how many hours per day were these machines used? I_II_I hours/days

And how many days per year I_II_II_I days/year

## As a worker in an oil refinery:

Were you involved in the production, extraction, or rectification of benzene? ** Yes  No**

Have you worked in tank servicing, equipment maintenance in refineries or fuel depots? ** Yes  No**

Have you worked at filling railcars and tank trucks? ** Yes  No**

Have you been involved in the production, extraction, rectification of benzene and products containing it? ** Yes  No**

Specify:

- The frequency of the task: I_II_I times per day/week/month/year *(circle)*
- Quantities handled I_II_II_I_I Liters per day/week/month/year *(circle)*
- Specify +++ Individual and collective means of protection ……………………………………………….

*………………………………………………………………………………………………………………………………………………..*

## Have you worked in the manufacture of intermediate synthetic chemicals such as: ethylbenzene, cumene, cyclohexane, nitrobenzene?

** Yes  No**

Specify:

- The frequency of the task: I_II_I times per day/week/month/year *(circle)*
- Quantities handled I_II_II_I_I Liters per day/week/month/year *(circle)*
- Specify +++ Individual and collective means of protection ……………………………………………….

*………………………………………………………………………………………………………………………………………………..*

## Have you worked in the perfume manufacturing industry? NB: exposure to benzène before 1995

** Yes  No**

Specify:

- Frequency of benzene use: I_II_I times per day/week/month/year *(circle)*
- Quantities handled I_II_II_I_I Liters per day/week/month/year *(circle)*
- Specify +++ Individual and collective means of protection ……………………………………………….

*………………………………………………………………………………………………………………………………………………..*

## Have you worked in the production of varnishes, paints, enamels, putties, inks, glues, cleaning products containing benzene?

** Yes  No**

Specify:

- Frequency: I_II_I times per day/week/month/year *(circle)*
- Quantities handled I_II_II_I_I Liters per day/week/month/year *(circle)*
- Specify +++ Individual and collective means of protection ……………………………………………….

*………………………………………………………………………………………………………………………………………………..*

## As a house painter:

**What product(s) do you use to clean your instruments?** *NB: White spirit containing benzene especially before 1980*

Specify:

- Frequency of cleaning: I_II_I times per day/week/month/year *(circle)*
- Quantities handled I_II_II_I_I Liters per day/week/month/year *(circle)*
- Specify +++ Individual and collective means of protection ……………………………………………….

*………………………………………………………………………………………………………………………………………………..*

**What product(s) do you use to clean your hands?** *NB: idem prior question*

Specify:

- Frequency of cleaning: I_II_I times per day/week/month/year *(circle)*
- Quantities handled I_II_II_I_I Liters per day/week/month/year *(circle)*
- Specify +++ Individual and collective means of protection ……………………………………………….

*………………………………………………………………………………………………………………………………………………..*

**What did you mainly use as paint?** *NB: Presence in glycerophthalic paint of petroleum derivatives, aromatic hydrocarbons (benzene, toluene, xylene).*

- Water-based paint ?  ** Yes  No**
- Peinture glycérophtalique?  ** Yes  No**

If yes for glycerophtalic paints: quantity used I_II_II_I_I Liters per day/week/month/year *(circle)*

**What diluent(s) did you use for your paints?** *NB: White spirit that may expose to benzene before 1980*

Specify:

- Frequency of use: I_II_I times per day/week/month/year *(circle)*
- Quantities handled I_II_II_I_I Liters per day/week/month/year *(circle)*
- Specify +++ Individual and collective means of protection ……………………………………………….

*………………………………………………………………………………………………………………………………………………..*

## As a textile worker, have you been involved in degreasing operations? NB: hydrocarbon solvents that may contain benzene before 1980  Yes  No

Specify:

- Frequency: I_II_I times per day/week/month/year *(circle)*
- Quantities handled I_II_II_I_I Liters per day/week/month/year *(circle)*
- Specify +++ Individual and collective means of protection ……………………………………………….

*………………………………………………………………………………………………………………………………………………..*

## As an electrician / electronic technician:

**What product(s) do you use to clean your instruments?** *White spirit ?*

*………………………………………………………………………………………………………………………………………………..*

Specify quantities handled I_II_II_I_I Liters per day/week/month/year *(circle)*

**What product(s) do you use to clean your hands?** *White spirit ? Other ? (Possible exposure to benzene before 1980)*

…………………………………………………………………………………………………………………………………………………………….

Specify quantities handled I_II_II_I_I Liters per day/week/month/year *(circle)*

## Have you worked in research, development, or analytical laboratories in the physical or natural sciences, or in teaching and handling benzene? NB: Benzene: restricted use since 1993 in professional and technological high schools   Yes  No

Specify:

- Frequency of handling: I_II_I times per day/week/month/year *(circle)*
- Quantities handled I_II_II_I_I Liters per day/week/month/year *(circle)*
- Specify +++ Individual and collective means of protection ……………………………………………….

*………………………………………………………………………………………………………………………………………………..*

## As a worker in the printing industry:

**What was the printing technique used?** (Typography, flexography, lithography, offset, photoengraving, gravure, screen printing...)

*NB: rotogravure: exposure to benzene ++ in light oil cuts before 1980 …………………………………………………………………………………………………………………………………………………………………..*

…………………………………………………………………………………………………………………………………………………

**What solvent(s) did you use for cleaning instruments and hands?** (E.g. aromatic solvents (benzene), naphtha solvents, special gasolines, petroleum) ? *NB: significant exposure to benzene contained in petroleum solvents before 1980)*

*……………………………………………………………………………………………………………………………………………………………*

If yes, specify:

- Frequency of cleaning: I_II_I times per day/week/month/year *(circle)*
- Quantities handled I_II_II_I_I Liters per day/week/month/year *(circle)*
- Specify +++ Individual and collective means of protection ……………………………………………….

*………………………………………………………………………………………………………………………………………………..*

## As an automobile and machinery mechanic

**Did you maintain vehicles running:**

- Gasoline only? ** Yes  No**
- Diesel fuel only? ** Yes  No**
- With both types? ** Yes  No**

if yes in which proportion? /____/ % gasoline et /______ / % diesel fuel

**What was the capacity in terms of vehicles in the garage?** (Number of simultaneous vehicles) /________ /

**What product(s) did you use to clean or degrease metal parts?** (e.g. White spirit? TCE? naphta? gasoline?) …………………………………………………………………………………………….

…………………………………………………………………………………………………………………………………………………………….

Quantities handled I_II_II_I_I Liters per day/week/month/year *(circle)*

Was the bin covered? ** Yes  No**

Was there a local aspiration? ** Yes** ** No**

**What product(s) did you use to clean your instruments?** Ex: White spirit? naphta?...................................................................................................................................................

Quantity used: /________ / Liters per day/week/month/year *(circle)*

**What product did you use to clean your hands?** *NB: White spirit: Presence de benzene before 1980*

*……………………………………………………………………………………………………………………………………………………………………………..*

How often did you clean your hands during the day? /________ / per day

How much did you use? /________ / Liters per day/week/month/year *(circle)*

## As an automotive painter, what type of paint did you use? NB. before 1980, presence of benzene …………………………………………………………………………………………………………………………………………

Volume used: /______ / Liters per day/week/month/year *(circle)*

Was there a paint room? ** Yes  No**

## Have you handled natural or synthetic rubber dissolvers or sharpening solvents? Before 1980, may contain benzene   Yes  No

Specify:

- Frequency of handling: I_II_I times per day/week/month/year *(circle)*
- Quantities handled I_II_II_I_I Liters per day/week/month/year *(circle)*
- Specify +++ Individual and collective means of protection ……………………………………………….

*………………………………………………………………………………………………………………………………………………..*

## As a metalworker, what product(s) did you use to clean your hands? White spirit could contain benzene before 1980

## ………………………………………………………………………………………………………………………

Specify:

- Frequency of handling: I_II_I times per day/week/month/year *(circle)*
- Quantities handled I_II_II_I_I Liters per day/week/month/year *(circle)*
- Specify +++ Individual and collective means of protection ……………………………………………….

*………………………………………………………………………………………………………………………………………………..*

- 1. **Formaldehyde questionnaire**

## Have you been involved in operations to sterilize eggs with formaldehyde?

** Yes  No**

How many interventions did you do each year? /_______ /

What volume (in liters) did you use each year? /_______ / Liters per year

## Did you use formaldehyde in the context of a farm?

** Yes  No**

How many interventions did you do each year? /_______ /

What volume (in liters) did you use each year? /_______ / Liters per year

## If you have worked in the agri-food sector:

Can you specify the nature of the food you were handling?

…………………………………………………………………………………………………………………………………………………………….

…………………………………………………………………………………………………………………………………………………………….

Have you participated in meat or fish smoking activities?

*Exposure to formaldehyde while operating the smoking facility*

** Yes  No**

If yes, please specify

- smoking frequency: I_II_I times a day/week/month/year (circle)
- How much time did you spend on it? /_______ / hours per day/week (circle)

## Were you involved in cleaning and disinfection operations of equipment and premises (food industry, hospital sector for disinfection of rooms or operating theatres? In the hospital environment, commonly done by nurses or orderlies until the 1990s. These products are still marketed in 2016

** Yes  No**

If yes, did you use formaldehyde as a fumigant? ** Yes  No**

Specify:

- Frequency of use: I_II_I per day/week/month/year (circle)
- Quantities used I_II_II_I_I Liters per day/week/month/year (circle)

Did you use any other substance(s) to disinfect? ** Yes  No**

Specify:

- Frequency of use: I_II_I per day/week/month/year (circle)
- Quantities used I_II_II_I_I Liters per day/week/month/year (circle)

Did any of them contain formaldehyde?**  Yes  No**

- Which ones?..................................................................................................................................
- Specify quantities used I_II_II_I_I Liters per day/week/month/year (circle)

## Did you work on the preparation of formaldehyde or its solutions (formalin)?

** Yes  No**

Specify:

- Frequency of handling: I_II_I times per day/week/month/year *(circle)*
- Quantities handled I_II_II_I_I Liters per day/week/month/year *(circle)*
- Specify +++ Individual and collective means of protection ……………………………………………….

*………………………………………………………………………………………………………………………………………………..*

## Have you work in the manufacture or use of phenol-formaldehyde, urea-formaldehyde or melamine-formaldehyde resins (e.g. varnishing and vitrification of floors and furniture, fabric finishing)?

** Yes  No**

Specify:

- Frequency of handling: I_II_I times per day/week/month/year *(circle)*
- Quantities handled I_II_II_I_I Liters per day/week/month/year *(circle)*
- Specify +++ Individual and collective means of protection ……………………………………………….

*………………………………………………………………………………………………………………………………………………..*

## Have you worked in the manufacture of disinfectant products containing formaldehyde?

** Yes  No**

Specify:

- Frequency of handling: I_II_I times per day/week/month/year *(circle)*
- Quantities handled I_II_II_I_I Liters per day/week/month/year *(circle)*
- Specify +++ Individual and collective means of protection ……………………………………………….

*………………………………………………………………………………………………………………………………………………..*

## Have you worked in the manufacture of end-use products using formaldehyde or a formaldehyde releaser as a preservative?

** Yes  No**

Specify:

- Frequency of handling: I_II_I times per day/week/month/year *(circle)*
- Quantities handled I_II_II_I_I Liters per day/week/month/year *(circle)*
- Specify +++ Individual and collective means of protection ……………………………………………….

*………………………………………………………………………………………………………………………………………………..*

## As a metalworker:

**Did you use cutting oils (whole oils or soluble oils)?**

*Exposure to formaldehyde used as such or release of formaldehyde through machining, but negligible quantity*

** Yes  No**

If yes, was the machine hooded? ** Yes  No**

If yes, specify:

- Frequency of handling: I_II_I times per day/week/month/year *(circle)*
- Quantities handled I_II_II_I_I Liters per day/week/month/year *(circle)*
- Specify +++ Individual and collective means of protection ……………………………………………….

*………………………………………………………………………………………………………………………………………………..*

**Did you use aminoplast or phenoplast resins for the manufacture of moulds in metallurgy?  Yes  No**

If yes, specify:

- Frequency of handling: I_II_I times per day/week/month/year *(circle)*
- Quantities handled I_II_II_I_I Liters per day/week/month/year *(circle)*
- Specify +++ Individual and collective means of protection ……………………………………………….

*………………………………………………………………………………………………………………………………………………..*

## Have you worked as an thanatopractor and used formaldehyde?

** Yes  No**

If yes, specify:

- Frequency of handling: I_II_I times per day/week/month/year *(circle)*
- Quantities handled I_II_II_I_I Liters per day/week/month/year *(circle)*
- Specify +++ Individual and collective means of protection ……………………………………………….

*………………………………………………………………………………………………………………………………………………..*

## Have you worked in a medical analysis or pathology laboratory? Exposure to formaldehyde, other solvents

** Yes  No**

Were you a formaldehyde user or did you directly handle parts treated with formaldehyde?**  Yes  No**

If yes, specify:

- Frequency of handling: I_II_I times per day/week/month/year *(circle)*
- Quantities handled I_II_II_I_I Liters per day/week/month/year *(circle)*

Has the intensity of your exposure been specified by atmospheric measurements?

** Yes  No**

If yes, can you give the results? ........................................................................

## Have you used formaldehyde as a disinfectant for reusable surgical instruments or probes?

** Yes  No**

If yes, specify:

- The form in which formaldehyde was used (name of the end-use products and how they were used) ……………………………………………………………………………………………………………………

…………………………………………………………………………………………………………………………………………………..

- Frequency of handling: I_II_I times per day/week/month/year *(circle)*
- Quantities handled I_II_II_I_I Liters per day/week/month/year *(circle)*
- Specify +++ Individual and collective means of protection ……………………………………………….

*………………………………………………………………………………………………………………………………………………..*

## Have you worked in the manufacture of particleboard, medium-density fibreboard, plywood, framing or glulam?

*NB: use of formaldehyde in urea-formaldehyde glues and binders, melanin-formaldehyde still used today.* ** Yes  No**

If yes, specify:

- Frequency of handling: I_II_I times per day/week/month/year *(circle)*
- Quantities handled I_II_II_I_I Liters per day/week/month/year *(circle)*
- Specify +++ Individual and collective means of protection ……………………………………………….

*………………………………………………………………………………………………………………………………………………..*

## Did you use or cut particleboard, medium density fiberboard, plywood, framing or glulam?

** Yes  No**

If yes, specify:

- Frequency of handling: I_II_I times per day/week/month/year *(circle)*
- Quantities handled I_II_II_I_I Liters per day/week/month/year *(circle)*
- Specify +++ Individual and collective means of protection ……………………………………………….

*………………………………………………………………………………………………………………………………………………..*

- 1. **Pentachlorophenol questionnaire**

*NB1: Ban on the use of pentachlorophenol since 1994, with an exemption until 2008 for wood preservation (treated materials must not be in contact with food products or have agricultural use).*

*NB2: Contamination of pentachlorophenol by PCBs and PCDDs*

## Have you worked in the manufacture of particleboard, medium-density fibreboard, plywood, framing or glulam?

*NB: Pentachlorophenol until 2008, which may be contaminated with dioxin.*

** Yes  No**

If yes, specify:

- Frequency of handling: I_II_I times per day/week/month/year *(circle)*
- Quantities handled I_II_II_I_I Liters per day/week/month/year *(circle)*
- Specify +++ Individual and collective means of protection ……………………………………………….

*………………………………………………………………………………………………………………………………………………..*

## Did you use pentachlorophenol for tissue treatment?

*Used in France until the early 1990s in the outdoor clothing sector and the maritime sector (sails, etc.).* ** Yes  No**

If yes, specify:

- Frequency of handling: I_II_I times per day/week/month/year *(circle)*
- Quantities handled I_II_II_I_I Liters per day/week/month/year *(circle)*
- Specify +++ Individual and collective means of protection ……………………………………………….

*………………………………………………………………………………………………………………………………………………..*

- 1. **Trichloroethylene questionnaire**

## If you worked as a house painter before 1980, did you use trichloroethylene to clean your hands or tools? Potential exposure before 1980

** Yes  No**

If yes, specify:

- Frequency of cleaning: I_II_I times per day/week/month/year *(circle)*
- Quantities handled I_II_II_I_I Liters per day/week/month/year *(circle)*
- Specify +++ Individual and collective means of protection ……………………………………………….

*………………………………………………………………………………………………………………………………………………..*

## Were you involved in the production, packaging or transport of TCE?  Yes  No

Specify the tasks carried out, the frequency of the stain (per month), the quantities handled (liters), the protective means worn, etc. ……………………………………………………………………………………………………..

…………………………………………………………………………………………………………………………………………………………..

## As an electrical/electronics worker prior to 1980, did you use trichloroethylene to clean your hands or tools?  Yes  No

If yes, specify:

- Frequency of use: I_II_I times per day/week/month/year *(circle)*
- Quantities handled I_II_II_I_I Liters per day/week/month/year *(circle)*
- Specify +++ Individual and collective means of protection ……………………………………………….

*………………………………………………………………………………………………………………………………………………..*

## As a worker in a dry cleaning facility, did you use trichloroethylene for dry cleaning or pre-spotting?  Yes  No

If yes, specify:

- Frequency of cleaning: I_II_I times per day/week/month/year *(circle)*
- Quantities handled I_II_II_I_I Liters per day/week/month/year *(circle)*
- Specify +++ Individual and collective means of protection ……………………………………………….

*………………………………………………………………………………………………………………………………………………..*

## As a metalworker, have you used trichloroethylene for hot or cold degreasing of metal parts, for example, in the surface metallization, stamping, bar turning or machining sectors? NB: TCE++ before the end of the 1970s

*TCE+++ before the end of the 1970s*

** Yes  No**

Did you use trichloroethylene for manual degreasing? ** Yes  No**

If yes, what quantities handled I_II_II_I_I Liters per day /week/month/year (circle)

Did you use trichloroethylene for a degreasing in a tank ** Yes  No**

If yes, specify if it was ** **cold or ** **hot

Was the bin covered? ** Yes  No**

Was there a local aspiration? ** Yes  No**

## As an automobile/machine mechanic, prior to 1980, did you use trichloroethylene to clean your hands or tools?  Yes  No

If yes, specify:

- Frequency of use: I_II_I times per day/week/month/year *(circle)*
- Quantities handled I_II_II_I_I Liters per day/week/month/year *(circle)*
- Specify +++ Individual and collective means of protection ……………………………………………….

*………………………………………………………………………………………………………………………………………………..*

- 1. **Dichloromethane (Methylene Chloride) questionnaire**
  2. **As a woodworker, did you use paint strippers for glycerophthalic paints and/or varnishes?**NB: presence of DCM in paint strippers for glycerol varnishes

If yes, specify:

- Frequency of use: I_II_I times per day/week/month/year *(circle)*
- Quantities handled I_II_II_I_I Liters per day/week/month/year *(circle)*
- Specify +++ Individual and collective means of protection ……………………………………………….

*………………………………………………………………………………………………………………………………………………..*

## Have you been involved in the stripping of facades, frame murals, shutters or blinds in the building or subcontracting companies?  Yes  No

If yes, specify:

- Frequency of use: I_II_I times per day/week/month/year *(circle)*
- Quantities handled I_II_II_I_I Liters per day/week/month/year *(circle)*
- Specify +++ Individual and collective means of protection ……………………………………………….

*………………………………………………………………………………………………………………………………………………..*

## As a house painter, did you use dichloromethane or paint strippers to clean your instruments?  Yes  No

If yes, specify:

- Frequency of use: I_II_I times per day/week/month/year *(circle)*
- Quantities handled I_II_II_I_I Liters per day/week/month/year *(circle)*
- Specify +++ Individual and collective means of protection ……………………………………………….

*………………………………………………………………………………………………………………………………………………..*

## Have you used methylene chloride for the production of cosmetic aerosols?

 Yes  No

If yes, specify:

- Frequency of use: I_II_I times per day/week/month/year *(circle)*
- Quantities handled I_II_II_I_I Liters per day/week/month/year *(circle)*
- Specify +++ Individual and collective means of protection ……………………………………………….

*………………………………………………………………………………………………………………………………………………..*

## Were you involved in the synthesis, packaging or transport of methylene chloride?

##  Yes  No

If yes, specify:

- Frequency of the task: I_II_I times per day/week/month/year *(circle)*
- Quantities handled I_II_II_I_I Liters per day/week/month/year *(circle)*
- Specify +++ Individual and collective means of protection ……………………………………………….

*………………………………………………………………………………………………………………………………………………..*

## Have you used methylene chloride in the manufacture of cellulosic fibres?

 Yes  No

If yes, specify:

- Frequency of use: I_II_I times per day/week/month/year *(circle)*
- Quantities handled I_II_II_I_I Liters per day/week/month/year *(circle)*
- Specify +++ Individual and collective means of protection ……………………………………………….

*………………………………………………………………………………………………………………………………………………..*

## Have you used methylene chloride in the leather industry?

 Yes  No

If yes, specify:

- Frequency of use: I_II_I times per day/week/month/year *(circle)*
- Quantities handled I_II_II_I_I Liters per day/week/month/year *(circle)*
- Specify +++ Individual and collective means of protection ……………………………………………….

*………………………………………………………………………………………………………………………………………………..*

## Have you used methylene chloride as extraction and process solvents in the food industry?  Yes  No

If yes, specify:

- Frequency of use: I_II_I times per day/week/month/year *(circle)*
- Quantities handled I_II_II_I_I Liters per day/week/month/year *(circle)*
- Specify +++ Individual and collective means of protection ……………………………………………….

*………………………………………………………………………………………………………………………………………………..*

## Did you use methylene chloride in the manufacture of textile stain removers?

 Yes  No

If yes, specify:

- Frequency of use: I_II_I times per day/week/month/year *(circle)*
- Quantities handled I_II_II_I_I Liters per day/week/month/year *(circle)*
- Specify +++ Individual and collective means of protection ……………………………………………….

*………………………………………………………………………………………………………………………………………………..*

## Have you used dichloromethane in the manufacture of printed circuit boards?

 Yes  No

If yes, specify:

- Frequency of use: I_II_I times per day/week/month/year *(circle)*
- Quantities handled I_II_II_I_I Liters per day/week/month/year *(circle)*
- Specify +++ Individual and collective means of protection ……………………………………………….

*………………………………………………………………………………………………………………………………………………..*

## Did you use dichloromethane for cold degreasing of metals?

 Yes  No

Did you use dichloromethane for manual degreasing? ** Yes  No**

If yes, what quantities handled I_II_II_I_I Liters per day /week/month/year (circle)

Did you use dichloromethane for degreasing in a tank? ** Yes  No**

If yes, was the bin covered? ** Yes  No**

Was there a local aspiration? ** Yes  No**

## Have you used methylene chloride in chemical analysis laboratories?

##  Yes  No

If yes, specify:

- Frequency of use: I_II_I times per day/week/month/year *(circle)*
- Quantities handled I_II_II_I_I Liters per day/week/month/year *(circle)*
- Specify +++ Individual and collective means of protection ……………………………………………….

*………………………………………………………………………………………………………………………………………………..*

## Have you used methylene chloride as an extraction and process solvent in the pharmaceutical industry?

##  Yes  No

If yes, specify:

- Frequency of use: I_II_I times per day/week/month/year *(circle)*
- Quantities handled I_II_II_I_I Liters per day/week/month/year *(circle)*
- Specify +++ Individual and collective means of protection ……………………………………………….

*………………………………………………………………………………………………………………………………………………..*

## As an automobile/machine mechanic:

**Have you used dichloromethane to clean or degrease metal parts?**

 Yes  No

**Did you use dichloromethane for manual degreasing?**  ** Yes  No**

If yes, what quantities handled I_II_II_I_I Liters per day /week/month/year (circle)

Did you use it for degreasing in a tank? ** Yes  No**

If yes, was the bin covered? ** Yes  No**

Was there a local aspiration? ** Yes  No**

## As an employee in the plastics industry, have you used dichloromethane as a blowing agent for polyurethane foams or as a polymerization solvent (e.g. polycarbonates...)?

##  Yes  No

If yes, specify:

- Type of use ……………………………………………………………………………………………………………………..

……………………………………………………………………………………………………………………………………………………

- Frequency of use: I_II_I times per day/week/month/year *(circle)*
- Quantities handled I_II_II_I_I Liters per day/week/month/year *(circle)*
- Specify +++ Individual and collective means of protection ……………………………………………….

*………………………………………………………………………………………………………………………………………………..*

- 1. **Polychlorobiphenyls (PCBs), Polychlorinated Dibenzodioxins and Polychlorinated Dibenzofurans (PCDD/F) questionnaire**

## Did you make or lay linoleum? NB: Presence of PCBs until 1987

 Yes  No

If yes, specify:

- Frequency of the task: I_II_I times per day/week/month/year (circle)

## Have you worked in the production, packaging or transport of PCBs?

 Yes  No

If yes, specify:

- Frequency of use: I_II_I times per day/week/month/year *(circle)*
- Quantities handled I_II_II_I_I Liters per day/week/month/year *(circle)*
- Specify +++ Individual and collective means of protection ……………………………………………….

………………………………………………………………………………………………………………………………………………

## Have you intervened on transformers or capacitors containing PCBs (pyralenes, arochlors, askarels, phenochlors)? NB: Presence of PCBs as an insulating fluid in transformers/condensers placed on the market before 1987 (date of prohibition of the sale, acquisition or placing on the market of equipment containing PCBs or products with more than 0.005% by weight). Transformers and capacitors are deemed to have been labelled to specify the presence or absence of these substances after 1987.

##  Yes  No  DK

If yes, specify:

- Frequency of use: I_II_I times per day/week/month/year *(circle)*
- Quantities handled I_II_II_I_I Liters per day/week/month/year *(circle)*
- Specify +++ Individual and collective means of protection ……………………………………………….

………………………………………………………………………………………………………………………………………………

## Did you use any paints, glues, inks or other materials that you knew or learned contained PCBs?

** Yes  No  DK**

If yes, specify:

- Nature of the materials ……………………………………………………………………………………………………..

……………………………………………………………………………………………………………………………………………………

- Frequency of use: I_II_I times per day/week/month/year *(circle)*
- Quantities handled I_II_II_I_I Liters per day/week/month/year *(circle)*
- Specify +++ Individual and collective means of protection ……………………………………………….

## Have you worked in the synthetic rubber manufacturing sector?

*NB: TCDD, but negligible exposure*

** Yes  No  DK**

If yes, specify:

- Frequency of the task: I_II_I times per day/week/month/year *(circle)*
- Quantities handled I_II_II_I_I Liters per day/week/month/year *(circle)*
- Specify +++ Individual and collective means of protection ……………………………………………….

………………………………………………………………………………………………………………………………………………

## Have you worked in the manufacture of rubber objects, such as tires or gloves? NB: TCDD

** Yes  No  DK**

If yes, specify:

- Frequency of the task: I_II_I times per day/week/month/year *(circle)*
- Quantities handled I_II_II_I_I Liters per day/week/month/year *(circle)*
- Specify +++ Individual and collective means of protection ……………………………………………….

………………………………………………………………………………………………………………………………………………

## Have you worked in the manufacturing of herbicides or wood treatment products, including herbicides derived from trichlorophenoxyacetic acid or 2,3,5 trichlorophenol (2,4,5-T, 2,4-D...) and pentachlorophenol? NB: PCCD and furans

** Yes  No  DK**

If yes, specify:

- The substances involved………………………………………………………………………………………………………..

……………………………………………………………………………………………………………………………………………………

- Frequency of handling: I_II_I times per day/week/month/year *(circle)*
- Quantities handled I_II_II_I_I Liters per day/week/month/year *(circle)*
- Specify +++ Individual and collective means of protection ……………………………………………….

………………………………………………………………………………………………………………………………………………

## Have you used herbicides derived from trichlorophenoxyacetic acid or 2,3,5 trichlorophenol (phenoxyherbicides: 2,4,5-T, 2,4-D... or chloronitrate derivatives) or wood treatment products containing pentachlorophenol?

** Yes  No  DK**

If yes, specify:

- The substances involved………………………………………………………………………………………………………..

……………………………………………………………………………………………………………………………………………………

- Frequency of handling: I_II_I times per day/week/month/year *(circle)*
- Quantities handled I_II_II_I_I Liters per day/week/month/year *(circle)*
- Specify +++ Individual and collective means of protection ……………………………………………….

………………………………………………………………………………………………………………………………………………

## Have you worked in the production of chlorobenzenes or chlorophenols?

** Yes  No  DK**

If yes, specify:

- The substances involved………………………………………………………………………………………………………..

……………………………………………………………………………………………………………………………………………………

- Frequency of handling: I_II_I times per day/week/month/year *(circle)*
- Quantities handled I_II_II_I_I Liters per day/week/month/year *(circle)*

Specify +++ Individual and collective means of protection ……………………………………….…… ………………………………………………………………………………………………………………………………………………

## Have you worked in the production or use of chlorinated dioxazine dyes? NB: TCCD contamination

** Yes  No  DK**

If yes, specify:

- The substances involved………………………………………………………………………………………………………..

……………………………………………………………………………………………………………………………………………………

- Frequency of handling: I_II_I times per day/week/month/year *(circle)*
- Quantities handled I_II_II_I_I Liters per day/week/month/year *(circle)*

Specify +++ Individual and collective means of protection ……………………………………….…… ………………………………………………………………………………………………………………………………………………

## Have you worked in the paper manufacturing sector, particularly during the pulp chlorination bleaching stage? NB: presence of TCDD

** Yes  No  DK**

If yes, specify:

- Frequency of the task: I_II_I times per day/week/month/year *(circle)*
- Quantities handled I_II_II_I_I Liters per day/week/month/year *(circle)*
- Specify +++ Individual and collective means of protection ……………………………………………….

………………………………………………………………………………………………………………………………………………

## Did you work as a plumber/maintenance technician on high-temperature circuits in the food industry or on ships transporting fuels before 1975?  Yes  No

If yes, specify:

- Frequency of the task: I_II_I times per day/week/month/year *(circle)*
- Quantities handled I_II_II_I_I Liters per day/week/month/year *(circle)*
- Specify +++ Individual and collective means of protection ……………………………………………….

………………………………………………………………………………………………………………………………………………

## Have you cleaned or maintained industrial or domestic incinerators, cement plant chimneys, etc.? NB: exposure to TCDD

** Yes  No  DK**

If yes, specify:

- Frequency of the task: I_II_I times per day/week/month/year *(circle)*
- Quantities handled I_II_II_I_I Liters per day/week/month/year *(circle)*
- Specify +++ Individual and collective means of protection ……………………………………………….

………………………………………………………………………………………………………………………………………………

- 1. **Butadiene questionnaire**

## Have you worked in the production, packaging or transport of butadiene??  Yes  No  DK

If yes, specify

- Circumstances of exposure …………………………………………………………………………………………………..

……………………………………………………………………………………………………………………………………………………

- Frequency of the task: I_II_I times per day/week/month/year *(circle)*
- Quantities handled I_II_II_I_I Liters per day/week/month/year *(circle)*
- Specify +++ Individual and collective means of protection ……………………………………………….

………………………………………………………………………………………………………………………………………………

## Have you worked in the manufacture of rubber objects, such as tires or gloves   Yes  No

If yes, specify

- Circumstances of exposure …………………………………………………………………………………………………..

……………………………………………………………………………………………………………………………………………………

- Frequency of the task: I_II_I times per day/week/month/year *(circle)*
- Quantities handled I_II_II_I_I Liters per day/week/month/year *(circle)*
- Specify +++ Individual and collective means of protection ……………………………………………….

………………………………………………………………………………………………………………………………………………

## Have you worked in the manufacture of ABS (acrylonitrile-butadiene-styrene) thermoplastic resins?   Yes  No

If yes, specify

- Circumstances of exposure …………………………………………………………………………………………………..

……………………………………………………………………………………………………………………………………………………

- Frequency of the task: I_II_I times per day/week/month/year *(circle)*
- Quantities handled I_II_II_I_I Liters per day/week/month/year *(circle)*
- Specify +++ Individual and collective means of protection ……………………………………………….

………………………………………………………………………………………………………………………………………………

## Have you worked in the manufacture of MBS (methacrylate-butadiene-styrene)??

** Yes  No**

If yes, specify

- Circumstances of exposure …………………………………………………………………………………………………..

……………………………………………………………………………………………………………………………………………………

- Frequency of the task: I_II_I times per day/week/month/year *(circle)*
- Quantities handled I_II_II_I_I Liters per day/week/month/year *(circle)*
- Specify +++ Individual and collective means of protection ……………………………………………….

………………………………………………………………………………………………………………………………………………

## Have you worked in the production of liquified petroleum gas, its packaging, transportation, distribution or have you been exposed to it by working on liquified petroleum gas motor vehicles? NB: use of butane containing high amounts of butadiene

** Yes  No**

If yes, specify

- Circumstances of exposure …………………………………………………………………………………………………..

……………………………………………………………………………………………………………………………………………………

- Frequency of the task: I_II_I times per day/week/month/year *(circle)*
- Quantities handled I_II_II_I_I Liters per day/week/month/year *(circle)*
- Specify +++ Individual and collective means of protection ……………………………………………….

………………………………………………………………………………………………………………………………………………

- 1. **Ethylene Oxide questionnaire**

## Have you worked in the production, packaging or transport of ethylene oxide?

 Yes  No

If yes, specify

- Circumstances of exposure …………………………………………………………………………………………………..

……………………………………………………………………………………………………………………………………………………

- Frequency of the task: I_II_I times per day/week/month/year *(circle)*
- Quantities handled I_II_II_I_I Liters per day/week/month/year *(circle)*
- Specify +++ Individual and collective means of protection ……………………………………………….

………………………………………………………………………………………………………………………………………………

## Have you participated in the sterilization of medical equipment with ethylene oxide or transported/stored equipment that has just been sterilized with ethylene oxide?

*Use of ethylene oxide only in industrial environments and not in health care facilities. The product had to desorb for a certain time, colored strips used as a tracer.*

 Yes  No

If yes, specify

- Circumstances of exposure …………………………………………………………………………………………………..

……………………………………………………………………………………………………………………………………………………

- Frequency of the task: I_II_I times per day/week/month/year *(circle)*
- Quantities handled I_II_II_I_I Liters per day/week/month/year *(circle)*
- Specify +++ Individual and collective means of protection ……………………………………………….

………………………………………………………………………………………………………………………………………………

## Have you used ethylene oxide for the manufacture of ethanolamines, nonionic surfactants or ethylene glycol?

 Yes  No

If yes, specify

- Circumstances of exposure …………………………………………………………………………………………………..
- Frequency of the task: I_II_I times per day/week/month/year *(circle)*
- Quantities handled I_II_II_I_I Liters per day/week/month/year *(circle)*
- Specify +++ Individual and collective means of protection ……………………………………………….

………………………………………………………………………………………………………………………………………………

- 1. **Ionizing Radiation questionnaire**

## Did you work in a medical imaging, interventional cardiology or interventional radiology department, or equivalent?  Yes  No

Specify the circumstances of exposure:……………………………………………………………………………………………….

…………………………………………………………………………………………………………………………………………………………….

Do you benefit from dosimetric monitoring? ** Yes  No**

If yes, indicate if it revealed any anomalies and if so, specify which ones …………………………………

…………………………………………………………………………………………………………………………………………………………….

## Have you worked in a nuclear medicine department, in a department for the preparation of radioactive chemical and/or pharmaceutical products? Radioisotopes (technetium 99m, iodine 131, strontium, gallium, thallium): gamma ray emitters

##  Yes  No

Specify the circumstances of exposure: ………………………………………………………………………………………………

Do you benefit from exposure monitoring? ** Yes  No**

If yes, indicate which one and if it revealed anomalies, specify which ones: ……………………………………

…………………………………………………………………………………………………………………………………………………………….

## Have you been exposed to sources of ionizing radiation in industry or in a laboratory?

##  Yes  No

Specify the circumstances of exposure:……………………………………………………………………………………………….

…………………………………………………………………………………………………………………………………………………………….

Do you benefit from dosimetric monitoring? ** Yes  No**

If yes, indicate if it revealed any anomalies and if so, specify which ones …………………………………

…………………………………………………………………………………………………………………………………………………………….

## Have you worked in the nuclear industry (extraction, manufacture, use or reprocessing of fuels, storage or treatment of nuclear waste)?

** Yes  No**

Specify the circumstances of exposure:……………………………………………………………………………………………….

…………………………………………………………………………………………………………………………………………………………….

Do you benefit from dosimetric monitoring? ** Yes  No**

If yes, indicate if it revealed any anomalies and if so, specify which ones …………………………………

…………………………………………………………………………………………………………………………………………………………….

## Have you participated in air atomic tests?  Yes  No

Specify the circumstances of exposure:……………………………………………………………………………………………….

…………………………………………………………………………………………………………………………………………………………….

Do you benefit from dosimetric monitoring? ** Yes  No**

If yes, indicate if it revealed any anomalies and if so, specify which ones …………………………………

…………………………………………………………………………………………………………………………………………………………….

## Have you worked in the extraction of uranium ores?  Yes No

## 2002: closure date of the last mine in France

Specify the circumstances of exposure:……………………………………………………………………………………………….

…………………………………………………………………………………………………………………………………………………………….

Do you benefit from dosimetric monitoring? ** Yes  No**

If yes, indicate if it revealed any anomalies and if so, specify which ones …………………………………

…………………………………………………………………………………………………………………………………………………………….

## Have you worked in the field of radiation disinfection or sterilization?  Yes  No

Specify the circumstances of exposure:……………………………………………………………………………………………….

…………………………………………………………………………………………………………………………………………………………….

Do you benefit from dosimetric monitoring? ** Yes  No**

If yes, indicate if it revealed any anomalies and if so, specify which ones …………………………………

…………………………………………………………………………………………………………………………………………………………….

## Have you performed metal mass detection in airports?

##   Yes  No

Specify the frequency and the duration of the exposure ……………………………………………………………….

…………………………………………………………………………………………………………………………………………………………….

## Have you used radioactive sources in measuring devices such as humidifiers, gamma-densitometers (measurement of soil density in construction), radiometric thickness or level gauges or ionizers?

** Yes  No**

Specify the circumstances of exposure:……………………………………………………………………………………………….

…………………………………………………………………………………………………………………………………………………………….

Do you benefit from dosimetric monitoring? ** Yes  No**

If yes, indicate if it revealed any anomalies and if so, specify which ones …………………………………

…………………………………………………………………………………………………………………………………………………………….

## Have you worked in the field of ion smoke detectors (also known as DFCI for ion chamber smoke detectors)?

## NB: Contain the radioelement americium.

Specify the circumstances of exposure to ionizing radiations:……………………………………………………….

…………………………………………………………………………………………………………………………………………………………….

Do you benefit from monitoring of exposure? ** Yes  No**

If yes, indicate if it revealed any anomalies and if so, specify which ones …………………………………

…………………………………………………………………………………………………………………………………………………………….

## Have you worked in the field of lead detection in paints?

## NB: Portable X-ray fluorescence spectrometer with radioactive sources

** Yes  No**

If yes, specify

- The frequency of exposure: I_II_I per hour per day/week/month/year (circle)
- The duration of exposure I_II_II_I day/week/month/year (circle)

**EVALUATION OF THE QUALITY OF THE QUESTIONNAIRE**

***FEASIBILITY OF THE QUESTIONNAIRE***

For the patient you have just interviewed, do you find this questionnaire easy to use?

** Yes  No**

If not, why? ……………………………………………………………………………………………………………………………………………………..

…………………………………………………………………………………………………………………………………………………………………………………………..

For the patient you just interviewed, how long did the questionnaire take? ................. minutes

***ACCEPTABILITY OF THE QUESTIONNAIRE***

For the patient you have just interviewed, do you judge this questionnaire:

 Easily acceptable

 Moderately acceptable

 Hardly acceptable

Any remarks to be made: …………………………………………………………………………………………………………………………………………………………………………………….

…………………………………………………………………………………………………………………………………………………………………………………….
