# Supplementary material for: Development of a Questionnaire for the Search for Occupational Causes in Patients with Non-Hodgkin Lymphoma: The RHELYPRO Study
Source: Int J Environ Res Public Health. 2021 Apr 11;18(8):4008. doi: 10.3390/ijerph18084008 (PMC8068898; doi:10.3390/ijerph18084008)
Supplement: Supplementary file 1 [file ijerph-18-04008-s001.zip › ijerph-1140628-FR-supplementary/Questionnaire S2_OHE Questionnaire_29mar21.docx]

**Occupational History Expert**

**Questionnaire (OHE)**

**EMPLOYMENT N° X**

*(To be completed for each job)*

Start Year: __ __ __ __ End Year: __ __ __ __

**Company name and address: _______________________________________**

**_______________________________________**

**_______________________________________**

**Main activity of the company: ______________________________________**

**______________________________________**

**Approximate number of employees:**  <10  10 . 49  50 – 249  > 250

**Job Title: ______________________________________**

Working time: Part time: __ __ hours/week Full time

Was it a night job or a 3x8 shift? Yes No

If yes, how many nights did you work each month?  

**Job description**

- Premises, machines, materials and products used (brands)
- Dust, fumes, gases and vapors at the workstation
- Individual or collective protection (cabin, hood, ventilation, aspiration...), glasses, gloves, masks,

clothing)

Work done by colleagues around your workstation? Have you ever replaced a colleague, if so for what task and for how long?

(Describe in detail the different tasks performed, starting with the main task and continuing with the other tasks). Report here the information provided by the general supplementary questions and the specific questionnaires, if any.

......................................................................................................................................

......................................................................................................................................

......................................................................................................................................

......................................................................................................................................

......................................................................................................................................

......................................................................................................................................

/

***NAF Code 2008***

***ILO Code 2008***

***Social Security System***

***1. General***

***2. Agriculture***

***3. Independent***

***4. Civil Service***

***5. Other***

..................................................................................................................................................................................................................................................................................................................................................................................................................................................................................................................................................................................................................................................................................................................................................................................................................................................................................................................................................................................................................................................................................................................................................................................................................................................................................................................................................................................................................................................................................................................................................................................................................................................................................................................................................................................................................................................................................................................................................................................................................................................................................................................................................................................................................................................................................................................................................................................................................................................................................................................................................................................................................................................................................................................................................................................................................................................................................................................................................................................................................................................................................................................................................................................................................................................................................................................................................................................................................................................................................................................................................................................................................................................................................................................................
